# A local uPAR-plasmin-TGF*β*1 positive feedback loop in a qualitative computational model of angiogenic sprouting explains the *in vitro* effect of fibrinogen variants

**DOI:** 10.1371/journal.pcbi.1006239

**Published:** 2018-07-06

**Authors:** Sonja E. M. Boas, Joao Carvalho, Marloes van den Broek, Ester M. Weijers, Marie-José Goumans, Pieter Koolwijk, Roeland M. H. Merks

**Affiliations:** 1 Centrum Wiskunde & Informatica (CWI), Amsterdam, The Netherlands; 2 Mathematical Institute, Leiden University, Leiden, The Netherlands; 3 CFisUC, Department of Physics, University of Coimbra, Coimbra, Portugal; 4 Amsterdam Cardiovascular Sciences, VU University medical Center, Dept. of Physiology, Amsterdam, The Netherlands; 5 Department of Cell and Chemical Biology, Leiden University Medical Center, Leiden, The Netherlands; University of California Irvine, UNITED STATES

## Abstract

In experimental assays of angiogenesis in three-dimensional fibrin matrices, a temporary scaffold formed during wound healing, the type and composition of fibrin impacts the level of sprouting. More sprouts form on high molecular weight (HMW) than on low molecular weight (LMW) fibrin. It is unclear what mechanisms regulate the number and the positions of the vascular-like structures in cell cultures. To address this question, we propose a mechanistic simulation model of endothelial cell migration and fibrin proteolysis by the plasmin system. The model is a hybrid, cell-based and continuum, computational model based on the cellular Potts model and sets of partial-differential equations. Based on the model results, we propose that a positive feedback mechanism between uPAR, plasmin and transforming growth factor *β*1 (TGF*β*1) selects cells in the monolayer for matrix invasion. Invading cells releases TGF*β*1 from the extracellular matrix through plasmin-mediated fibrin degradation. The activated TGF*β*1 further stimulates fibrin degradation and keeps proteolysis active as the sprout invades the fibrin matrix. The binding capacity for TGF*β*1 of LMW is reduced relative to that of HMW. This leads to reduced activation of proteolysis and, consequently, reduced cell ingrowth in LMW fibrin compared to HMW fibrin. Thus our model predicts that endothelial cells in LMW fibrin matrices compared to HMW matrices show reduced sprouting due to a lower bio-availability of TGF*β*1.

## Author summary

Therapies for a range of medical conditions, including cancer, wound healing and diabetic retinopathy can benefit from a better control over the growth of blood vessels. The chemical properties of fibrin, the material that forms scabs in wounds and can also occur in large concentrations in tumors, can regulate the degree of blood vessel growth (angiogenesis). Angiogenesis can be mimicked in cell cultures. These allow us to modulate the chemical properties of fibrin and study the effect on angiogenesis. Fibrin occurs in high molecular weight (HMW) and in low molecular weight (LMW) forms. Interestingly, there is more ingrowth of angiogenic-like structures into HMW than in LMW fibrin, but the mechanisms are poorly understood. To get more insight into these, we constructed a computational model. Using the model, we propose and analyse a hypothetical mechanism for sprouting that could explain the differences in endothelial cell sprouting in LMW and HMW fibrin matrices. Our model suggests that cells digest fibrin, thus creating space for ingrowth. At the same time, digestion frees growth factors bound to fibrin, that activates further secretion of digestive enzymes by the cells. We propose that the resulting positive feedback loop spontaneously selects cells in the endothelial monolayer for ingrowth and helps the blood vessel sprout move deeper into the fibrin. This could be a complementary mechanism to lateral-inhibition by Delta-Notch for the selection of leader cells, also called ‘tip cells’. Our model predicts that endothelial cells in LMW fibrin compared to HMW fibrin show reduced sprouting due to a lower bio-availability of TGF*β*1.

## Introduction

Tissues that are low in oxygen stimulate the outgrowth of side-branches from nearby blood vessels, in a process called neo-angiogenesis. A detailed understanding of angiogenesis is relevant for a range of physiological and pathological processes where obtaining a fine-level control of angiogenesis is of interest. Pathologies such as poor wound healing or diabetic retinopathy will benefit from simulating angiogenesis, whereas inhibition (or tempering) angiogenesis is required in the treatment of tumors. Tissue engineering of large organs will require the growth of a functioning blood vessel system.

In wounds and in some tumors, new blood vessels are formed within fibrin matrices. Fibrin is formed as a provisional scaffold by leakage and subsequent polymerization of fibrinogen within the tissue. To form a new blood vessel endothelial cells (ECs) from nearby blood vessels invade this fibrin matrix. A suitable *in vitro* model for angiogenesis within fibrin was introduced by Koolwijk *et al*. [1]. In this model, a monolayer of human microvascular endothelial cells (hMVECs) is seeded on top of a layer of polymerized fibrin. When stimulated with a pro-angiogenic factor, such as vascular endothelial growth factor (VEGF) and/or basic fibroblast growth factor (bFGF), in combination with the inflammatory mediator TNF*α* (tumor necrosis factor *α*), endothelial sprouts grow into the fibrin matrix.

This hMVEC-fibrin system is in wide use as an assay to screen for stimulators and inhibitors of angiogenesis. However, in absence of an exact understanding of how known molecular and cellular mechanisms interlock to produce the observed, dynamic angiogenesis-like behavior, it is difficult to go much beyond a ‘trial and error’ approach and use the model system to rationally design new strategies for interfering with angiogenesis. By ‘reconstructing’ a cell culture model *in silico*, mathematical modeling provides insight into how known mechanisms work together and interlink to produce the observed behavior. This paper introduces a mathematical modeling approach to analyse, (a) what mechanisms regulate the onset of an angiogenic sprout (or ‘ingrowth spot’) in an endothelial cell monolayer, and (b) what mechanisms consolidate the further invasion of an angiogenic sprout. Together, these two observables determine the level of angiogenesis, making them relevant targets for tissue engineering and design of medical therapies.

### Fibrinogen variants

**Fig 1:**
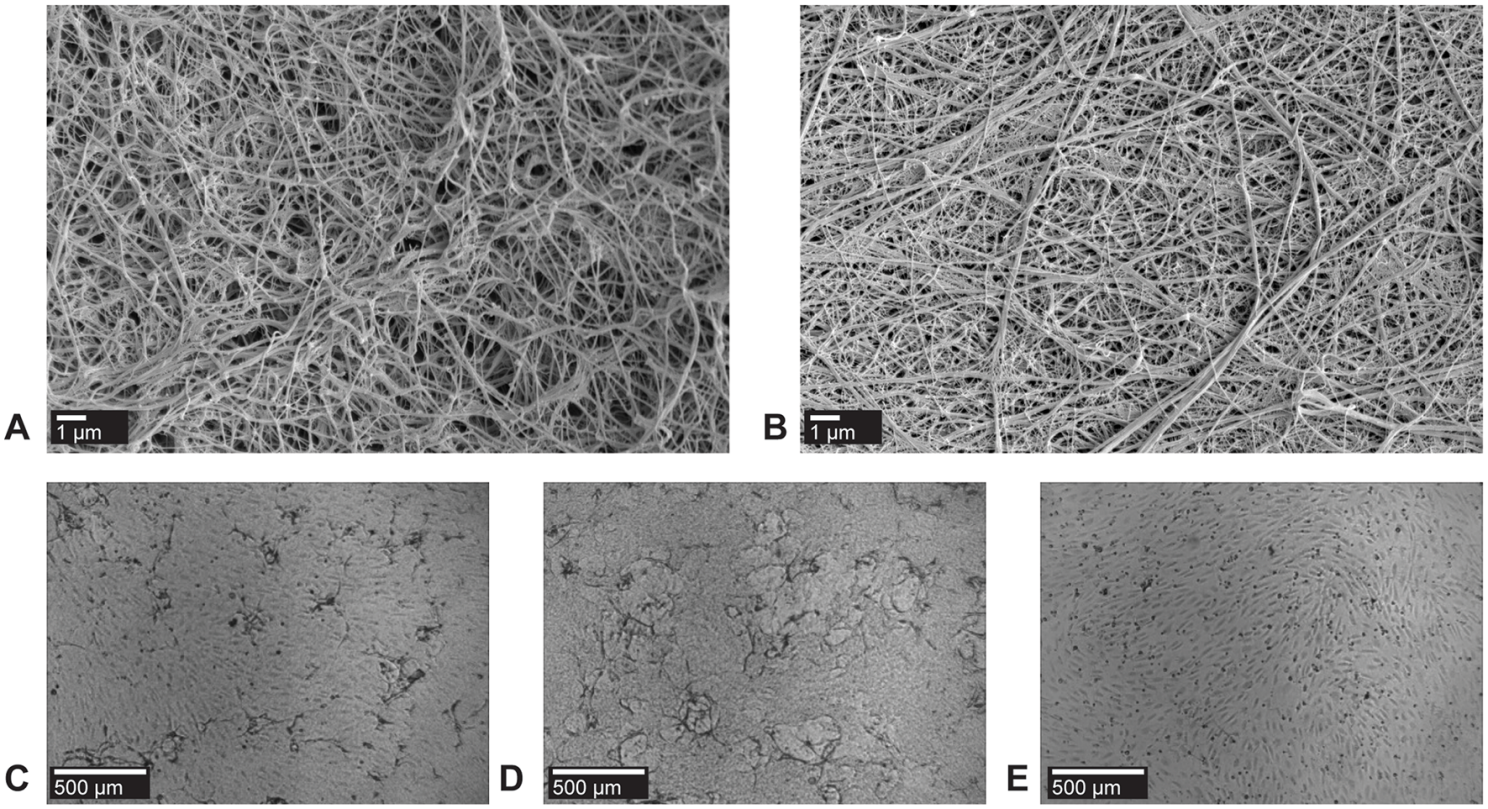
In vitro angiogenesis on HMW and LMW fibrin matrices. (A) Scanning electron microscopic analysis of a HMW fibrin network and (B) of a LMW fibrin network. The high molecular weight (HMW) form of fibrin has a more open network structure than the low molecular weight (LMW) form of fibrin, which has denser fibers [3]. Bars represent 1 *μ*m. (C) Representative top views of vascular ingrowth in unfragmented fibrin; (D) in HMW fibrin; and (E) in LMW fibrin. Experiments were performed as previously described [1]; independent replicates given in S2 Fig. Bars represent 500 *μ*m.

**Fig 2:**
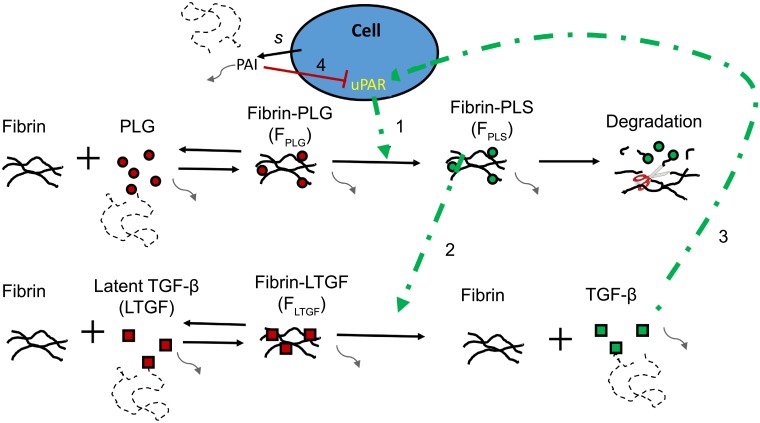
Schematic overview of plasmin and TGF*β*1 interactions. Plasminogen (PLG) reversibly binds fibrin, forming fibrin-bound plasminogen (*F*_PLG_). *F*_PLG_ is converted by cell-bound uPAR (arrow 1) to fibrin-bound plasmin (*F*_PLS_). *F*_PLS_ degrades fibrin. Latent-TGF*β*1 (LTGF) binds fibrin reversibly. Fibrin-bound latent-TGF*β*1 (*F*_LTGF_) is activated and released by *F*_PLS_ (arrow 2), resulting in active, diffusive TGF*β*1 and free fibrin. Active TGF*β*1 induces production of uPAR (arrow 3). Cells secrete (*s*) PAI-1 (PAI), which inhibits uPAR activity (arrow 4). The gray, dotted lines indicate diffusion of proteins and curved, gray lines indicate decay.

Fibrinogen occurs in three variants, whose relative abundance in fibrin affects its structure and pro-angiogenic capacity: (1) High molecular weight (HMW, MW 340 kDa, ∼70%, Fig 1A); (2) a low molecular weight form (LMW, MW 305 kDa, ∼26% of total fibrinogen, Fig 1B) that is formed after partial degradation of the carboxy-terminus of the fibrinogen A*α*-chain; and (3) an alternative low-molecular weight form (LMW’) that is formed after degradation of both A*α*-chains (MW 270 kDa, ∼4% of total fibrinogen) [2]. HMW fibrin has a more open matrix structure, with larger openings between the fibers compared to LMW fibrin (Fig 1A and 1B). LMW fibrin forms more complex networks with denser fibers. Fibrinogen composition is a key determinant of the number of ingrowth spots (Fig 1C–1E and S2 Fig) and the depth of sprouting [2–5]. hMVECs proliferate more and show more angiogenic ingrowth in HMW fibrin than in LMW or an unfractionated fibrin mixture [2].

The relative abundance of the three fibrinogen variants is changed in a number of pathologies. For example, the relative abundance of LMW and LMW’ has been found to be elevated in patients with vascular occlusion [6] and in patients with diabetes mellitus [7], possibly due to enhanced vascular leakage. In cancer patients the fibrinogen levels were elevated, but no changes in the HMW:LMW ratio were found [6]. In post-operative patients [6, 8] as well as after extensive acute myocardial infarction [8] the levels of HMW increased, followed by a delayed increase of LMW-fibrin [8]. In full-term newborns the levels of HMW have been found to be 25% lower than in adults [9].

Altogether, our *in vitro* evidence suggests that HMW-fibrinogen promotes angiogenesis more than LMW-fibrinogen. *In vivo*, increased levels of HMW are typically found in post-operative patients and after extensive myocardial infarction. It is unknown whether these changes in HMW:LMW ratios have clinical relevance, *e.g*., in stimulating angiogenesis (high HMW) in post-operative patients or in the inhibition (high LMW) of angiogenesis in diabetes mellitus patients.

### Fibrinolysis by the plasmin system

During angiogenic ingrowth, the invading hMVEC proteolytically digest the fibrin matrix, suggesting that the low efficiency of *in vitro* angiogenesis in LMW fibrin is due to differential regulation of proteolysis. Cell-associated fibrinolysis is mostly performed by the trypsin-like protease plasmin [10–13]. Plasmin is the active conversion product of plasminogen, which is mainly produced by the liver and reaches fibrin scaffolds through the blood stream. Conversion of plasminogen into plasmin occurs by plasminogen activators and is highly regulated. Urokinase-type plasminogen activator (uPA) and tissue-type plasminogen activator (tPA) are secreted by ECs as inactive single-chain proteins. tPA is expressed in quiescent endothelium [14] and is primarily involved in clot dissolution [15], whereas uPA and its cellular receptor (uPAR) are expressed during angiogenesis and control pericellular proteolysis [14, 16]. ECs secrete inactive, single chain pro-uPA that binds to uPA receptors (uPARs) on the membrane of endothelial cells, and is subsequently converted into an active two-chained form. This active membrane-bound uPA-uPAR complex converts plasminogen into plasmin [11]. To balance fibrin degradation, ECs secrete plasminogen inhibitor type 1 (PAI-1) that binds to tPA and uPA for deactivation and the PAI-1-uPA-uPAR complex is internalized [10, 12]. Alongside plasmin, membrane-type 1 metalloproteinase (MT1-MMP) can perform cell-associated fibrinolysis [17], although its role is still poorly understood: the MT1-MMP inhibitor TIMP-1 had only minor effects on sprouting in a 100% fibrin matrix, but was inhibiting when a 90% fibrin-10% collagen matrix was used [18]. Altogether, based on the available evidence we assume that hMVEC-associated fibrinolysis [2] is primarily due to the plasminogen-plasmin degradation system.

### Regulation of angiogenesis through release of latent-TGF*β*1

To get more insight into a potential role of fibrinogen variants in regulating angiogenesis, here we ask, using mathematical modeling, what differences between HMW and LMW fibrinogen could explain the differences in angiogenic ingrowth that are observed *in vitro*. LMW fibrin has a reduced number of binding sites for growth factors, including latent-TGF*β*1 [19]. TGF*β*1 has a strong pro-angiogenic effect in hMVEC cultured on Matrigel [20] and is present in latent form in fibrin matrices. Thus, apart from the structural differences between fibrin variants discussed above, a possible difference between fibrin HMW and LMW matrices is their binding capacity of TGF*β*1.

TGF*β*1 upregulates PAI-1 and uPAR and is inhibited by TGF*β*1 antagonist peptides. TGF*β*1 also induces PAI-1 and uPAR expression in hepatic stellate cells [21] and uPA/PAI-1 levels in human tumor tissues [22]. LTBP1 (latent transforming growth factor *β* binding protein 1) potentially binds the C-terminus of this A*α*-chain: LMW fibrinogen has a reduced number of C-termini of the A*α*-chain compared to HMW fibrinogen. The level of LTBP1 is dramatically reduced in LMW fibrinogen fraction I-9, compared to commercially available fibrinogen and intact fibrinogen fraction I-2 [19]. LTBP1 sequesters latent-TGF*β*1 in the plasma to fibrin, resulting in an inactive TGF*β*1 reservoir within the fibrin matrix that can locally be activated and released by plasmin [23–25]. Endothelial cells also secrete TGF*β*1 [25]; we here assume that this TGF*β*1 fraction can be neglected relative to the high bio-availability of TGF*β*1 in the matrix. Thus, the reduced number of LTBP1 binding sites in LMW fibrinogen compared to HMW fibrinogen can result in a lower bio-availability of TGF*β*1, thereby reducing angiogenesis.

### Mathematical modeling of fibrin invasion

Based on the experimental data on cell-associated fibrinolysis and TGF*β*1 that we have discussed above, we suggest that a local uPAR-plasmin-TGF*β*1 positive feedback loop drives angiogenesis (see Fig 2). For simplicity, we assume that all cell-bound uPAR is active, i.e., it is bound to uPA. Cell-bound uPAR activates plasmin (Fig 2, arrow 1) and plasmin locally degrades fibrin and releases and activates TGF*β*1 from its latent binding protein (see Fig 2, arrow 2). TGF*β*1 stimulates the production/expression of uPAR in the protruding cell (see Fig 2, arrow 3), whereas nearby cells, which experience only low TGF*β*1-dependent uPAR stimulation, are silenced by self-secreted PAI-1 (see Fig 2, arrow 4). The basic principle underlying this hypothesis is a *reinforced random walk* [26], as introduced to the problem of angiogenesis previously [27, 28]: (1) an external growth factor activates endothelial cells to enzymatically modify the ECM near the sprout, and (2) the endothelial cells move randomly, but with preference up gradient of the modified ECM.

More recent models have described specifically the hMVEC-fibrin culture system *in silico* [29, 30]. In both these previous models, the location of the novel capillary sprouts vascular ingrowths was specified a priori, prohibiting their use for analyzing the ‘degree’ of angiogenesis, usually measured as the number ingrowth spots in a cell culture [1]. Therefore, a detailed understanding and analysis of angiogenesis in the Koolwijk *et al*. [1] experimental 3D fibrin sprouting model requires two modifications of the assumptions in the previous work. Firstly, it is unpredictable which cells in the monolayer become sprout leaders (‘tip cells’). Thus we cannot pre-assign the location of the tip cells [29, 30], or assign the onset of angiogenesis by punching a hole in the basal lamina [31] or by initiating its local digestion [27, 28]. Also, previous models assumed that endothelial cells follow a gradient of VEGF. The Koolwijk *et al*. [1]*in vitro* model does not include growth factor gradients, so we have not included those in the present *in silico* model. This implies that both the location and the growth direction of sprouts in the present computational model emerge from local cell-cell and cell-matrix interactions. We hypothesize that such sprout initiation mechanisms may exist alongside the established role of the Dll4-Notch network in the selection of tip cells that lead the sprouts [32–35].

Altogether, to explore our hypothesis that the uPAR-plasmin-TGF*β*1 positive feedback loop regulates spontaneous ingrowth, we model the plasminogen-plasmin degradation system in combination with a cell-based model of endothelial cell invasion. following previous continuum models [36–38]. We propose that a differential binding activity of TGF*β*1 to HMW and LMW explains the higher ingrowth. We developed a computational model to evaluate if this sprouting mechanism can explain the reduced ingrowth in LMW compared to HMW; it is shown that it regulates the spacing of ingrowth spots and is also consistent with a number of additional observations.

## Results

### A computational model representing an *in vitro* 3D-fibrin sprouting model

**Fig 3:**
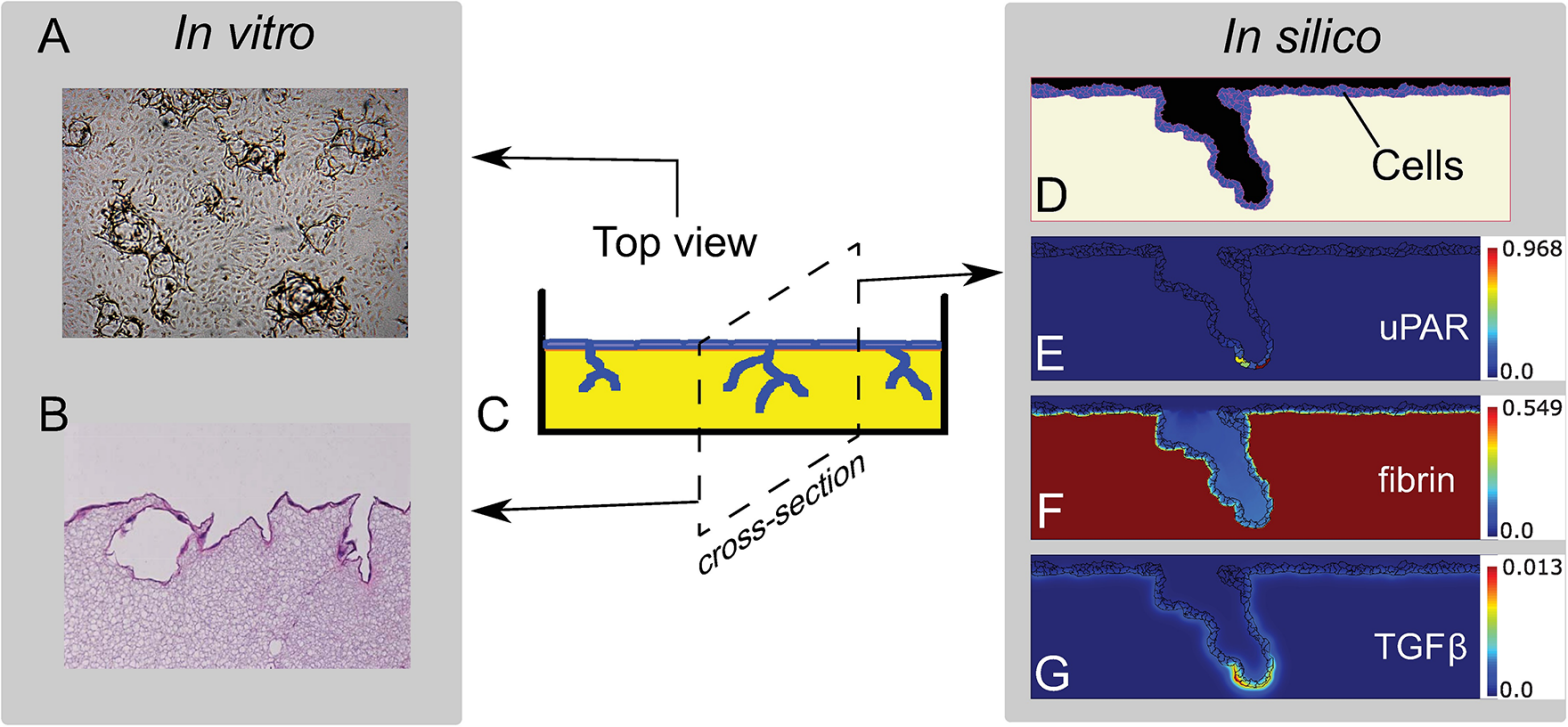
*In vitro* and *in silico* model setup. The *in vitro* model of Koolwijk *et al*. [1] can be studied with phase contrast views of the monolayer as used throughout this paper (A) or with cross-sections of the matrix after fixation and histological staining (B; see, e.g., Ref. [1, 39]; not used in this paper). A schematic illustration of the *in vitro* model (C) is shown in the middle, with a monolayer of endothelial cells (blue) that form capillary-like tubes in a fibrin matrix (yellow). Images of an *in silico* simulation that represents a cross-section of the *in vitro* model are shown on the right. Endothelial cells and fibrin (D) are modeled with the CPM, the uPAR concentration of cells (E) is modeled with an ODE equation, and a PDE system represents the concentrations of all forms of fibrin (F) and TGF*β*1 (G).

To study how endothelial sprouting is reduced in LMW compared to HMW fibrin matrices, we developed a computational model that mimics the *in vitro* assay by Koolwijk *et al*. [1] and Weijers *et al*. [2]. Our hybrid model consists of a cell-based component to describe the endothelial cells and a continuum component to describe the plasminogen-plasmin system. The model represents a cross-section of the *in vitro* sprouting model (Fig 3), and is initialized with a monolayer of fifty endothelial cells on top of a fibrin matrix. Fibrin forms a physical obstruction for cells while, at the same time, offering support to the cells as they can adhere to fibrin. Using cell-based modeling, we explicitly describe cell shape, cell motility, cell-cell adhesion, and cell-fibrin adhesion. Each cell has a level of active uPAR, which homogeneously spread over the cell membrane, and each cell secretes PAI-1 into the extracellular space. PAI-1, and the other extracellular proteins (fibrin, latent-TGF*β*1, active TGF*β*1, plasminogen, and plasmin) are modeled as concentration fields. The extracellular proteins interact with one another and with the membrane-bound uPAR (Fig 2). uPAR activates plasminogen, forming plasmin that degrades fibrin and locally activates latent-TGF*β*1 by releasing it from the fibrin matrix. The active TGF*β*1 induces the production of uPAR in nearby cells. These reactions form a local positive feedback loop that keeps the invasion of the endothelial cells going.

To represent cells and their physical interactions with the fibrin matrix, the cellular Potts model (CPM) [40, 41] was used. For details see Section Fibrin invasion. Briefly, the CPM represents cells on a regular lattice as patches of connected lattice sites. Cells move by copying lattice sites inward or outward, representing the extension and retraction of pseudopodia. To model the physical obstruction imposed by high concentrations of fibrin, the extension probability of a pseudopodium is reduced if it attempts to invade a lattice site with high fibrin concentration. The concentration of fibrin, $f(\vec{x})$, is initialized at a uniform, non-dimensional concentration of $f(\vec{x})=1.0$ at all lattice sites $\vec{x}$. No fibrin is produced or added in the simulation, such that the concentration of fibrin will stay in the range $f\left(\vec{x}\right)\in \left[0,1\right]$. The invasion probability quickly drops for concentrations $f(\vec{x})>0.5$, while for $f(\vec{x})<0.3$ fibrin does not hinder invasion.

Fibrin is digested by the plasmin system, as illustrated in Fig 2. The concentration of uPAR within each cell is modeled by an ordinary differential equation (ODE). A concentration field for uPAR is projected on the CPM grid, with each lattice site that is occupied by a cell having the uPAR concentration of that cell. The concentration of uPAR moves along with the location of the cell after cell movement. A system of coupled partial differential equations (PDEs, see Section Methods) describes the reactions between fibrin, TGF*β*1, plasminogen, plasmin, PAI-1 and all fibrin-bound forms. The equations for the plasmin system were based on the continuum model by Diamond *et al*. [36], which studies the penetration of uPA and tPA into a fibrin clot in the blood stream. To adopt this model to our problem, we added the uPAR-plasmin-TGF*β*1 positive feedback loop, simplified the implementation of fibrinolysis, and deleted the convective terms.

Time steps in our model are measured as Monte Carlo step (MCS). One MCS is defined as the number of lattice site update attempts as there are sites in the lattice. It takes about 6000 MCS to simulate a 10 days long experimental assay, similar to the 3D-fibrin sprouting model of Koolwijk *et al*. [1]; so a MCS represents approximately 2.5 minutes in real time.

In summary, the model is based on the following mechanistic assumptions:

1. Cell-bound uPA carries out active proteolysis. We do not consider the activity of t-PA, because addition of tPA specific antibodies does not have a significant effect on the formation of capillary-like tubular structures in the *in vitro* 3D-fibrin sprouting model by Koolwijk *et al*. [1].
2. Plasminogen binds fibrin reversibly; binding of plasminogen to ECs is not included in the model.
3. Fibrin-bound plasminogen is converted to fibrin-bound plasmin by uPAR. Plasminogen is in a closed configuration in circulation, but binding to fibrin induces an open configuration that is much more susceptible for activation [42–44].
4. Plasmin remains fibrin-bound. As a result, plasmin is localized at the cell surface or in immediate proximity of the cell [45].
5. Endothelial cells secrete PAI-1, which diffuses and decays [1, 11].
6. PAI-1 inhibits uPAR-bound uPA activity by internalization of uPAR-PAI-1 complexes [10, 12].
7. Latent-TGF*β*1 binds fibrin reversibly [25], thereby implicitly model binding of LTBP1 to latent-TGF*β*1.
8. We assume a non-competitive binding of latent-TGF*β*1 and plasminogen to fibrin.
9. Fibrin-bound latent-TGF*β*1 is released and activated by plasmin, resulting in diffusive TGF*β*1 [25].
10. TGF*β*1 induces expression of uPAR [20].
11. At initialization the plasminogen and latent-TGF*β*1 are bound to fibrin. We assume that plasminogen and latent-TGF*β*1 are already bound to plasma-derived fibrin or are present in the serum and bind during the preparation of the fibrin matrix.

### uPAR-plasmin-TGF*β*1 positive feedback selects ‘uPAR-rich’ cells in the monolayer

In the *in vitro* 3D-fibrin sprouting assay by Koolwijk *et al*. [1], uroplasmin (uPA) and its receptor uPAR were localized specifically at the invading endothelial cells that lead the sprouts [46]. The selection mechanism of these ‘uPAR-rich’ cells in the monolayer is not fully understood. We therefore asked if the uPAR-plasmin-TGF*β*1 positive feedback mechanism is sufficient to confine uPAR-expression to the invasive cells.

**Fig 4:**
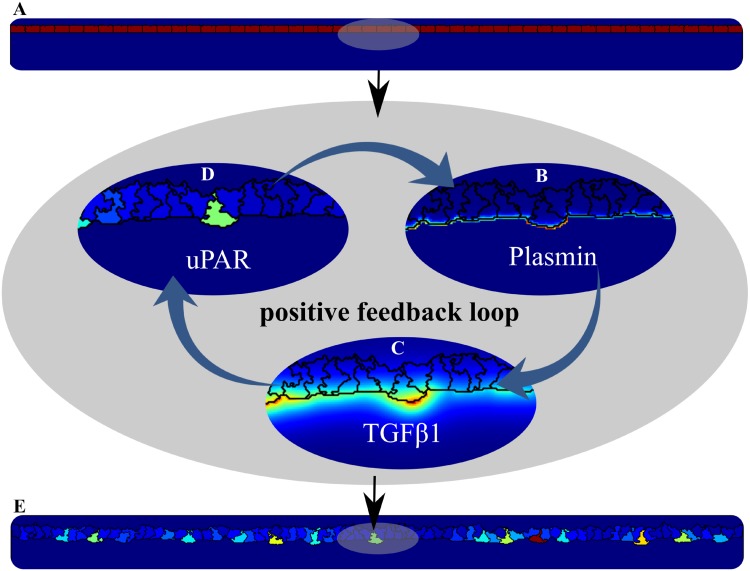
Spontaneous ‘uPAR-rich’ cell selection in the monolayer by a uPAR-plasmin-TGF*β*1 positive feedback loop. All cells in the model (A) express the same level of uPAR (the uPAR concentration in the cells is indicated by the red color) at initialization of a simulation. Local changes in fibrin-cell contact can increase local plasmin concentration (B), resulting in degradation of fibrin and release of active TGF*β*1 (C). TGF*β*1 can stimulate uPAR expression (D). The positive feedback loop selects ‘uPAR-rich’ cells in the monolayer (E), with a few cells having high level (red color) and most cells having low levels (blue color).

We initialized the cells in our model with a uniform concentration of uPAR (Fig 4A). Random cell movements change the contact-level and contact-duration with fibrin, resulting in local, random differences in the levels of plasmin activation. Fibrin is degraded at sites with a high plasmin activity (Fig 4B), and TGF*β*1 is released from the matrix (Fig 4C). The active TGF*β*1 induces the expression of uPAR in nearby cells (Fig 4D). The expression of uPAR in more distant cells can also be induced by the released TGF*β*1 to some degree, but such uPAR activity is counteracted by the self-secreted PAI-1. Due to stochasticity, only a few cells in the monolayer can trigger the positive feedback loop sufficiently to overcome inhibition by PAI-1 and gain high levels of uPAR to start ingrowth (Fig 4E). In absence of fibrin-bound latent-TGF*β*1, none of the cells in the monolayer, in a hundred stochastic simulations, manage to gain high levels of uPAR due to the lack of TGF*β*1-induced uPAR expression. Thus, our modeling results show that a uPAR-plasmin-TGF*β*1 positive feedback loop suffices to select uPAR-rich cells in a monolayer of endothelial cells to form ingrowth spots.

### uPAR-plasmin-TGF*β*1 positive feedback consolidates sprout progression

**Fig 5:**
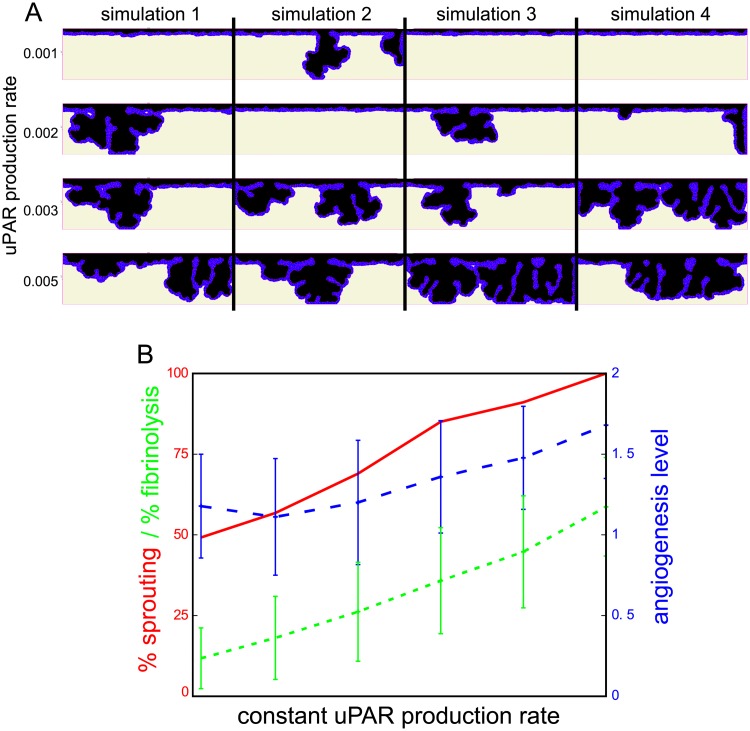
The angiogenesis level is sensitive to uPAR expression levels. Panel A shows a set of four stochastic simulation results at Monte Carlo step (MCS) 6000 for uPAR expression levels of 0.001, 0.002, 0.003, and 0.005 (RU/MCS). Sprouts form more frequently and more extensively at higher uPAR expression levels. For all simulations that formed sprouts, the mean angiogenesis level (blue curve) is calculated, representing the sprout count and sprout depth. The red curve in (B) represents the percentage of simulations that formed sprouts at MCS 6000 out of 100 simulations. The green curve in (B) represents fibrinolysis, the mean (out of 100 simulations) percentage of the initial fibrin lattice sites that are invaded by the endothelial cells at MCS 6000. Error bars are the standard deviation of 100 runs.

Once uPAR-rich cells are selected spontaneously within the monolayer, the uPAR-plasmin-TGF*β*1 positive feedback consolidates sprout progression in the model (see Fig 5). The cell leading the sprout, *i.e*., the *tip cell*, has the highest concentration of uPAR (see Fig 3E) in agreement with experimental observations [46].

In agreement with *in vitro* observations, in the *in silico* model sprouts branch spontaneously (see, *e.g*., simulation 3 for a constant uPAR production rate of 0.003 Relative Units (RU)/MCS in Fig 5A). This occurs when a cell adjacent to the tip cell moves into another direction, or when a cell higher up in the sprout manages to trigger the feedback loop and starts a branch. Sprouts are not formed in every simulation: due to the stochastic fluctuations in cell shape and movement, in some cases none of the cells activate the positive feedback loop sufficiently to overcome the inhibition of PAI-1. Similarly, ingrowth is not seen in every *in vitro* experiment, but is highly variable per cell donor and passage number of the cells within the same donor.

In vitro, TNF*α* is required to induce sprouting of human endothelial cells [1] and the mean tube length increases at higher doses of TNF*α*. TNF*α* increases uPA production and the level of cell-bound uPA [1]. To test if the model correctly reproduced this *in vitro* observation, we mimicked the effect of TNF*α* by increasing uPAR expression in the endothelial cells. Fig 5A shows a set of simulation results after ten days of sprouting for a uPAR expression level of 0.001, 0.002, 0.003, and 0.005 (RU/MCS). In simulations with higher uPAR expression levels, the number of ingrowth spots increases. For each parameter setting, four simulation results for the same parameter settings are shown; these demonstrate the stochasticity of ingrowth frequency and sprout morphology.

To quantify sprouting, we defined three measures: the angiogenesis level, the sprouting frequency and the fibrinolysis level. The angiogenesis level simultaneously reflects sprout depth and sprout count (see Section Methods for the quantification algorithm). The blue curve in Fig 5B represents the mean angiogenesis level for all simulations that formed sprouts (angiogenesis level>0). The sprouting frequency is the number of simulations out of a hundred simulations that formed sprouts (red curve in Fig 5B). The fibrinolysis level, defined as the mean percentage of initial fibrin lattice sites that are invaded by the endothelial cells in all 100 simulations, also increases for higher uPAR expression levels, as is expressed by the green curve in Fig 5B.

In summary, an increase of the basal uPAR-bound uPA activity in all cells increases the probability that the uPAR-plasmin-TGF*β*1 positive feedback loop is triggered in one of the cells in the monolayer, leading to high uPAR expression and sprout initiation. As a consequence, sprouts form more frequently and more excessively at higher uPAR expression levels. Thus, the model explains mechanically how ubiquitous stimulation of uPAR-bound uPA activity by TNF*α* leads to confined uPA activity and sprouting.

### Qualitative model validation

A full quantitative validation of the model is not feasible at present, because only for a few parameters experimental estimates are available, leaving most other parameters as fitting parameters. To avoid overfitting, we have instead selected a set of ‘default’ parameter values for which the model qualitatively reproduces the fibrin culture system (see S2 Table). To validate the model, we then tested if qualitative shifts in the parameter values, corresponding with published experiments, qualitatively reproduce the outcome of three published *in vitro* experiments of the plasminogen-plasmin degradation system.

**Fig 6:**
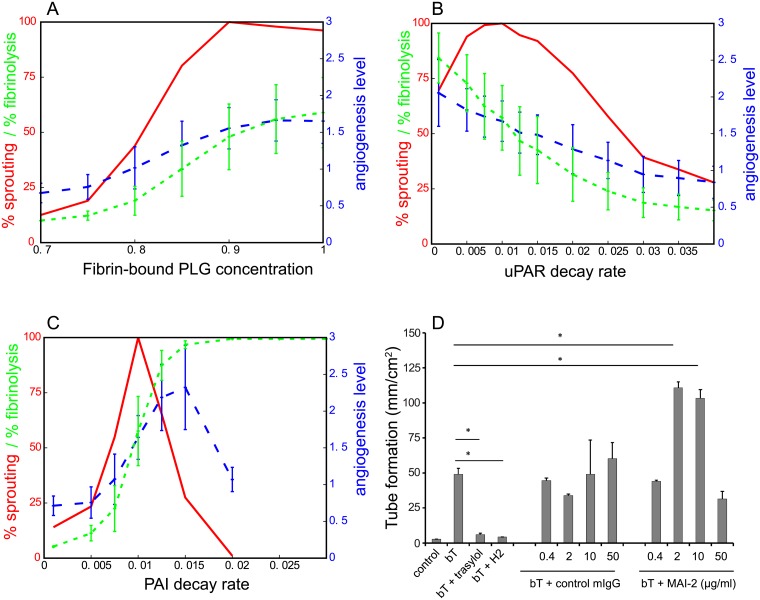
Model validation experiments. The sprouting percentage (red curve), the angiogenesis level (blue curve), and the fibrinolysis percentage (green curve), are plotted against changes in (A) the initial concentration of fibrin-bound plasminogen (relative units), (B) the decay rate of uPAR (MCS^−1^), and (C) the decay rate of PAI-1 (MCS^−1^). The sprouting percentage is the percentage of simulations (out of a 100 simulations) that have an angiogenesis level larger than zero. The angiogenesis level is a measure that simultaneously reflects sprout depth and sprout count, and the mean angiogenesis level is calculated over all simulations that actually formed sprouts. The fibrinolysis percentage is the percentage of the initial fibrin lattice sites that are invaded by the endothelial cells at MCS 6000. (D) Blocking PAI-1 activity increased endothelial sprouting in 3D fibrin matrices in a biphasic manner. hMVECs were seeded confluently on top of 3D fibrin matrices. Subsequently, the hMVECs were stimulated with the combination of FGF-2/TNF*α* (bT) with or without 100 U/ml trasylol, 25 ug/ml anti-uPAR antibody H2, control mIgG or anti-PAI-1 antibody MAI-2 (n = 4 independent donors, each in triplicate). 7 days after seeding and stimulation with FGF-2/TNF*α*, tube length was quantified by using Optimas software and expressed as mm/cm^2^ with error bars expressing standard error of the mean. For statistical analysis a one-way ANOVA with Bonferroni post-hoc test was used. * indicates P < 0.05. Error bars of panels A-C are the standard deviation of 100 runs.

Firstly, Koolwijk *et al*. [1] reported that there was no angiogenic ingrowth and tubule formation in fibrin matrices that were made using plasminogen-depleted fibrinogen. In agreement with this observation, there is no ingrowth in our model for low initial level of fibrin-bound plasminogen (Fig 6A). The sprouting percentage, the fibrinolysis percentage, and the angiogenesis level all increased with the initial fibrin-bound plasminogen concentration.

Secondly, inhibition of uPAR-bound uPA activity by addition of uPA specific polyclonal antibodies, or prevention of the binding of uPA to uPAR by soluble uPAR or blocking antibodies inhibited capillary-like tube formation dose-dependently (see Refs. [1, 46] and Fig 6D (bt + trasylol and bt + H2)). We mimicked the inhibition of uPAR activity by increasing the decay rate of uPAR. Consistent with the experimental results, Fig 6B shows that this parameter change results in a decrease of the sprouting percentage, the fibrinolysis percentage, and the angiogenesis level.

Thirdly, experiments show that there is an optimum PAI-1 concentration for angiogenesis [47]: addition of PAI-1 to implants in wild-type mice enhanced angiogenesis up to 3-fold at low concentrations but inhibited angiogenesis nearly completely at high concentrations. In the 3D fibrin assay, addition of the anti-PAI-1 antibody MAI-2 shows a similar biphasic effect on angiogenesis (Fig 6D): Moderate inhibition enhances tube formation, whereas strong inhibition reduces tube formation. This is due to excessive fibrinolysis, which is incompatible with normal capillary formation [48, 49]. As for uPAR, we modeled the manipulation of PAI-1 activity by an increase of the decay rate of PAI-1. Fig 6C shows that the fibrinolysis percentage strongly increases when the decay rate of PAI-1 is increased. High decay rate of PAI results in low PAI-1 activity, and thus in excessive fibrinolysis; no sprouts are formed, but the entire monolayer lowers simultaneously. Low decay rates of PAI-1 result in high PAI-1 activity and sprouting is completely inhibited. Only for intermediate levels of PAI-1 activity we find sprouting, indicated by the peaks in Fig 6C for the sprouting percentage and the angiogenesis level.

In conclusion, the model can reproduce three essential validation experiments for the plasminogen-plasmin system. In absence of fibrin-bound latent-TGF*β*1, no sprouts are formed in all 100 simulations with a parameter set for which sprouts formed well in presence of fibrin-bound latent-TGF*β*1 in Figs 5B and 6 (constant uPAR production rate = 0.005 RU/MCS, initial fibrin-bound plasminogen concentration = 1 RU, PAI-1 decay rate = 0.01 MCS^−1^, and uPAR decay rate = 0.0095 MCS^−1^, using Relative Units, RU, and Monte Carlo Steps, MCS). This shows that initialization and consolidation of sprouts can be driven by activity of the proposed positive feedback loop formed by uPAR, plasmin, and TGF*β*1.

### The bio-availability of TGF*β*1 regulates the level of endothelial sprouting in HMW and LMW fibrin matrices

Next we used our model to design new hypotheses about the mechanisms that reduce the level of angiogenic ingrowth in LMW fibrin matrices compared to HMW matrices. The level of LTBP1 is dramatically reduced in LMW fibrinogen fraction I-9, which lacks major parts of the C-termini of the A*α*-chain, compared to commercially available fibrinogen and intact fibrinogen fraction I-2 [19]. As LTBP1 sequesters latent-TGF*β*1 to fibrin, this could result in a lower level of fibrin-bound latent-TGF*β*1. We hypothesize that this reduced level of fibrin-bound latent-TGF*β*1, in combination with our suggested local uPAR-plasmin-TGF*β*1 positive feedback, could cause the reduced level of endothelial sprouting in LMW compared to HMW fibrin matrices. If the levels of inactive TGF*β*1 in the fibrin matrix are too low, cells are not able to induce a strong enough uPAR-plasmin-TGF*β*1 positive feedback loop to overcome the inhibition of PAI-1 and thus will not form sprouts.

**Fig 7:**
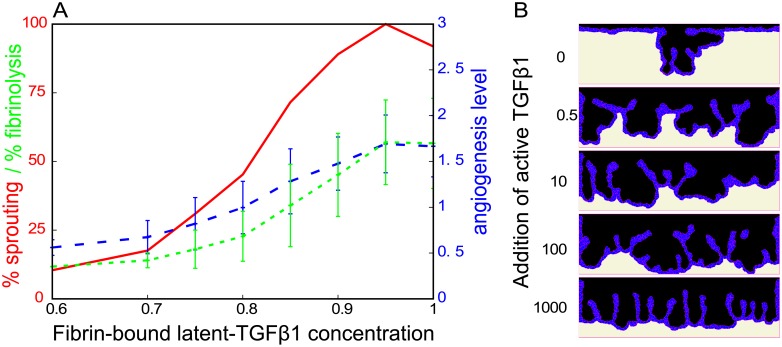
TGF*β*1 experiments. (A) The sprouting percentage (red curve), the angiogenesis level (blue curve), and the fibrinolysis percentage (green curve), are plotted against changes in the initial concentration of fibrin-bound latent-TGF*β*1 (relative units). The sprouting percentage is the percentage of simulations (out of a 100 simulations) that have an angiogenesis level larger than zero. The angiogenesis level is a measure that simultaneously reflects sprout depth and sprout count, and the mean angiogenesis level is taken over all simulations that actually formed sprouts. The fibrinolysis percentage is the percentage of the initial fibrin lattice sites that are invaded by the endothelial cells at MCS 6000. (B) Addition of active TGF*β*1 has a biphasic effect on sprout formation in our model. The sprouting frequency increases for the addition of low doses of TGF*β*1, but global degradation of the complete endothelial cell monolayer prevents sprout formation at high doses of TGF*β*1. Error bars are the standard deviation of 100 runs.

In line with this hypothesis, Fig 7A shows that the sprouting percentage, the fibrinolysis percentage, and the angiogenesis level decrease with lower initial concentrations of fibrin-bound latent-TGF*β*1 in our model. In conclusion, our simulations results suggest that the angiogenic ingrowth is reduced in LMW fibrin matrices compared to HMW matrices due to a reduction in binding sites for LTBP1.

The addition of active TGF*β*1 has a biphasic effect on *in vitro* sprouting [50]. Addition of active TGF*β*1 to the assay stimulates sprouting at low doses and inhibits sprouting at high doses of TGF*β*1. To test this biphasic effect in the model, we initialized the model with a homogeneously spread concentration of active TGF*β*1. The medium containing TGF*β*1 was refreshed every two days *in vitro* [50]. We similarly reset the TGF*β*1 concentration to the initial value after every two days in the model. Fig 7B shows that TGF*β*1 indeed has the reported biphasic effect on angiogenesis in the simulations. At low concentrations of added TGF*β*1 (TGF*β*1 = 0.5 RU and TGF*β*1 = 10 RU in Fig 7B), more sprouts are formed than without addition of TGF*β*1 (TGF*β*1 = 0 in Fig 7B). The uPAR-bound uPA activity in all cells increases due to the overall addition of TGF*β*1, allowing some cells to overcome the inhibitory PAI-1 threshold for triggering the uPAR-plasmin-TGF*β*1 positive feedback loop. This is a similar effect as was seen for the stimulation with TNF*α* above. The upregulation of uPAR-bound uPA activity is too strong at high doses of TGF*β*1, and consequently all cells degrade the matrix. This results in lowering of the complete endothelial cell monolayer, rather than in local sprouting (TGF*β*1 = 1000 RU in Fig 7B). In this case, fibrin is quickly degraded and some cells loose contact with the fibrin. Once the cells loose contact with the fibrin layer, they are no longer stimulated to migrate along with the degrading matrix and form the ‘fingers’ show in Fig 7B. In some simulations stacks of cells hovering above the monolayer were left behind. This is of course a model artifact, so we did not take those into account while quantifying the degree of spouting.

## Discussion

We have developed a computational model to study what mechanisms cause angiogenic ingrowth and subsequent sprouting in an *in vitro* model [1, 2] of angiogenic-like tubule formation of endothelial cells in 3D-fibrin matrices. For this purpose, we asked what mechanisms cause a reduced level of endothelial sprouting in low molecular weight (LMW) compared to high molecular weight (HMW) fibrin matrices [2]. We propose that a uPAR-plasmin-TGF*β*1 positive feedback loop selects ‘uPAR-rich’ cells and drives the further invasion of the sprout.

The mathematical model makes a number of plausible, mechanistic predictions on Koolwijk’s angiogenesis assay. Firstly, the model correctly predicts a reduced level of angiogenesis in LMW compared to HMW fibrin, suggesting that in LMW matrices the uPAR-plasmin-TGF*β*1 positive feedback loop is not activated. This could be due to LMW fibrin’s reduced binding capacity for TGF*β*1 [19]. Secondly, the model predicts that the uPAR-plasmin-TGF*β*1 positive feedback loop is responsible for the spontaneous selection of uPAR-expressing cells in the monolayer. Random cell movement activates the feedback loop more strongly in some cells than in others, resulting in random selection of sprout leader cells (tip cells) in the monolayer, and a large variability in the number of sprouts that are formed. Similarly, there is a large variation in the success of sprouting *in vitro*. Thirdly, and in line with this prediction, the base expression level of uPAR regulates the density of ‘uPAR-rich’ cells and sprouts. A possible candidate for the induction of uPAR-bound uPA activity is TNF*α* [1]. Addition of TNF*α* is required in the *in vitro* experiment with human endothelial cells to induce sprouting. Thus, our simulations provide an explanation for how upregulation of uPAR-bound uPA activity by TNF*α* can induce sprouting.

### Role of PAI-1 in sprout spacing

Endothelial cells in the *in vitro* assay of Koolwijk *et al*. [1] secrete PAI-1, but it is unknown if all cells, or only the quiescent cells in the monolayer or perhaps only the invading uPAR-rich cells secrete PAI-1. Interestingly, the uPAR-plasmin-TGF*β*1 positive feedback loop resembles reaction-diffusion systems with activator-inhibitor dynamics [51–53]. Activator-inhibitor systems produce periodic patters, so such dynamics could be responsible for regular placement of tip cells in the *in vitro* assay. Most conditions for activator-inhibitor dynamics are met: The positive feedback loops stimulates local activation of uPAR-bound uPA, and the inhibitor PAI-1 diffuses faster than the “activator” uPAR, which is expressed intracellularly. A missing element for such activator-inhibitor dynamics is that the inhibitor (PAI-1) must be produced locally, whereas we currently assumed that all cells secrete PAI-1. We are unsure of this assumption: TGF*β*1 induces production of uPAR as well as PAI-1 in MVEC cultured on Matrigel [20], raising the possibility that uPAR-rich cells secrete most PAI-1 and that all conditions for activator-inhibitor dynamics are met. Thus future work should determine the localization of PAI-1 secretion in the 3D-fibrin sprouting assay [1].

Besides the activator-inhibitor dynamics, the closely related substrate-depletion model [52] is a well-studied theoretical model for pattern formation. In our model, plasminogen is the substrate for plasmin production. Conversion of plasminogen at sites of matrix invasion results in depletion of plasminogen in surrounding regions through diffusion. Indeed, plasminogen is a limiting factor for endothelial sprouting in the fibrin assay [1]. Plasminogen depletion has low impact in the current simulations, because we have initialized them with a high, homogeneous concentration of immobile, fibrin-bound plasminogen. However, plasminogen binds fibrin reversibly and can bind to ECs, so this mechanism might regulate the location of ingrowth spots for lower levels of fibrin-bound plasminogen. Interestingly, there is a delay in sprout initiation when the model is initialized with unbound plasminogen. It takes some time to reach high enough concentrations of fibrin-bound plasminogen, which is then converted to plasmin by uPAR for matrix degradation.

### Interaction with Delta-Notch signaling

A key patterning mechanism that is involved in angiogenesis is lateral inhibition by Delta-Notch signaling [32–35]. Cells that have high levels of Delta ligands on their membrane differentiate into so called ‘tip cells’, which are the leaders of sprouts, and cells with low levels of Delta become ‘stalk cells’ [35]. Lateral inhibition occurs by interaction of Delta ligands with the Notch receptor of neighboring cells, resulting in the suppression of Delta production in those neighbors [32–35]. Lateral inhibition creates a pepper-and-salt pattern of tip and stalk cells, with tip cells surrounded by a rosette of stalk cells in monolayers *in silico* [54, 55]. Thus, Delta-Notch signaling alone cannot account for the more widely spaced pattern of uPAR-rich leader cells in a monolayer as observed *in vitro* [46]. Possibly other regulation mechanisms, *e.g*., the proposed uPAR-plasmin-TGF*β*1 positive feedback loop, act alongside the Delta-Notch mechanism to distribute tip cells more sparsely. Notably, gene expression levels of Dll4 and Notch4 are significantly higher in endothelial cells cultured in LMW matrices than in HMW matrices [2]. The Dll4 and Notch4 expression differences by themselves cannot explain the reduced ingrowth in LMW fibrin matrices, as specific inhibition of Dll4-Notch was unable to induce recovery of tube formation in LMW.

Inclusion of Delta-Notch signaling will likely affect sprout morphology. In simulations of our current model, cells adjacent to the tip cell are also activated by the released TGF*β*1, and they contribute to sprouting. This results in fairly wide, sometimes cyst-like sprouts. In our previous model [29] of the fibrin assay, narrow sprouts formed if only the tip cell secreted proteolytic enzymes for matrix degradation, and cyst-like sprouts formed when the stalk cells contributed to fibrin degradation as well. In this light, Delta-Notch signaling could repress proteolytic activity in cells adjacent to the tip cell, such that thinner sprouts will form.

### Open problems and future work

Our model explains differences in ingrowth between LMW and HMW fibrin based on the binding capacity of latent-TGF*β*1. An alternative explanation for the increased ingrowth in HMW fibrin compared to LMW fibrin could be that the ECs can invade the open matrix structure of HMW fibrin more easily. In absence of proteolysis, differences in matrix porosity can explain cell migration speed and persistence [56]; however, with small pore sizes of fibrin (order 1 *μm*; see Fig 1) and the importance of fibrinolysis for angiogenic ingrowth, small differences in pore size are unlikely to contribute to differences in ingrowth. An alternative, or complementary explanation could lie in differences in the bulk mechanical properties of HMW and LMW fibrin. Indeed mechanical cell-cell communication [57, 58] through strain-stiffening materials such as fibrin [59] suffices for generating vascular-like patterns [60]. In addition, individual fiber architecture, including fiber thickness and fiber density also affects cell spreading behavior on fibrin substrates independently of the bulk mechanical properties [61], suggesting that fiber architecture differences (Fig 1) could also contribute to differences in angiogenesis level on HMW and LMW fibrin matrices.

The present model reproduces angiogenic sprouting by means of cell-fibrin adhesion and cell-division. A limitation is that the addition of TNF*α* in the *in vitro* model inhibits cell division [1]. The general cell invasion mechanism proposed here does not depend on cell division. Alongside the fibrinolysis-driven sprouting mechanisms proposed here, many alternative mechanisms of cell migration during angiogenic sprouting have been proposed that could act alongside or instead of cell division to replenish cells in growing sprouts. A range of models have shown that mutual attraction of endothelial cells suffices for the formation of vascular networks, *e.g*., via a chemoattractant [62–70], via mechanical forces [71, 72] or via mechanically induced durotaxis [60], and preferential attraction to elongated structures [73]. Our model could be extended with such sprouting and cell migration mechanisms to replace cell division.

A detailed description of the plasminogen-plasmin system is included in our model, but still some simplifications were made. For instance, we did not take into account interactions with matrix metalloproteinases (MMPs). Membrane-type 1 metalloproteinase (MT1-MMP) can perform cell-associated fibrinolysis [17], but only plays a minor role in Koolwijk’s assay [18]. Furthermore, we neglected the low proteolytic activity of pro-uPA [11], and only modeled active uPAR-bound uPA. Interactions between pro-uPA and plasmin could give interesting dynamics. Venkatraman *et al*. [37] considered a positive feedback loop in which the initial cleavage of plasminogen into plasmin is more efficient by uPA than pro-uPA, and the conversion of pro-uPA to uPA is driven by plasmin. By the use of a continuum model, they predict that uPA-plasmin activation is bistable in the presence of this positive feedback loop in combination with substrate competition for plasmin.

A further limitation of the present model of the plasmin system, is that the numerical method cannot describe the advection of chemical species due to displacement of fibrin. This approximation is reasonable in the low Péclet number regime simulated here; *i.e*., cell movement (and the resulting advection of chemicals due to movement of fibrin) is much slower than the movement of chemicals relative to the ECM due to diffusion and fibrin degradation. Because cells cannot ‘push’ fibrin, but only grow over it if fibrin is sufficiently degraded, the low Péclet number regime is ensured for fibrin and all fibrin bound growth factors. Also, cell movement is slower than the diffusive spread of the unbound growth factors, further justifying our approximation. A suitable method for modeling advective transport in the CPM due to cell movement for higher Péclet number cases has been proposed elsewhere [74], and can be applied in future extensions of our model.

It could be argued that the present two-dimensional approximation *in silico* does not represent Koolwijk’s three-dimensional cell culture model well, because in two-dimensional cell cultures the cellular micro-environment is usually not well represented [75]. However, note that in two-dimensional cross-section the cellular micro-environment of the endothelial cells corresponds with those in the three-dimensional cell culture. The leading cell is flanked by other endothelial cells and by the fibrin matrix (see, e.g., the uPAR-rich cell in Fig 4D), whereas the following endothelial cells are flanked by fibrin, culture fluid and cells (see, e.g., Fig 5A). Thus the two-dimensional cross-section *in silico* suffices as an approximation of the three-dimensional model *in vitro*. Nevertheless, the model will run in 3D with some adjustments, through appropriate scaling of the cell volume constraint and the adhesion parameters [76]. The positive feedback loop hypothesis, and the mechanisms involved, will both work in the same qualitative way in 3D as in 2D, since the reaction-diffusion equations have the same form.

In conclusion, our model predicts that the reduced level of endothelial sprouting in LMW compared to HMW fibrin matrices can, at least in part, be explained by a reduced level of fibrin-bound latent-TGF*β*1 in LMW fibrin. To validate this hypothesis experimentally, we propose to check if there is indeed a reduced level of fibrin-bound latent-TGF*β*1 in the experimental setup [1, 2]. As a second experiment, we propose to validate whether sprouting can be reduced in HMW fibrin matrices by addition of TGF*β*1-antagonists. These validation experiments can bring us closer to an understanding of the mechanisms of selection of leader or ‘tip cells’ in the monolayer and sprouting in the *in vitro* setup.

## Methods

We developed a hybrid, cell-based and continuum, computational model of angiogenic sprouting to represent the *in vitro* 3D-fibrin sprouting assay of Koolwijk *et al*. [1] (Fig 3). The model includes a uPAR-plasmin-TGF*β*1 positive feedback loop that drives sprouting and is used to explain the reduced ingrowth on LMW compared to HMW. Cells and their physical interaction with fibrin are modeled with the cellular Potts model. The CPM is coupled to concentration fields to model the uPAR-plasmin-TGF*β*1 positive feedback loop. Each cell has a concentration of uPAR, homogeneously spread on its membrane, modeled by an ordinary differential equation (ODE). A system of partial differential equations (PDEs) describes the interactions between fibrin, plasminogen, plasmin, PAI-1 and TGF*β*1.

### Cellular Potts model

The shape and motility of endothelial cells are modeled with the cellular Potts model (CPM) [40, 41]. The model domain is a two-dimensional regular lattice $\Lambda \subset Z^{2}$, with $\vec{x}\in \Lambda$ the coordinates of the lattice sites. Cells and extracellular materials are projected onto the grid as patches of (usually connected) lattice sites, marked with the same unique identifier $\sigma (\vec{x})$. Thus a generalized cell (*e.g*., a cell or ECM material) *s* is defined as the set of lattice sites marked with the same identifier $\sigma (\vec{x})$, $C\left(s\right)=\{\vec{x}\in \Lambda |\sigma \left(\vec{x}\right)=s\}$. Each identifier is further associated with a type *τ*(*σ*). Here *τ*(*σ*) ∈ {cell, fibrin, cell, patch, border, medium}; its function is simply to define parameters and properties for categories of Potts domains, not for all domains individually.

Cells move by extending or retracting pseudopodia, which include lamellipodia, filopodia and invadopodia. Pseudopodia movement is modeled by attempting to copy the state ($\sigma (\vec{x})$) of a randomly selected lattice site $\vec{x}$ into a lattice site $\vec{x}\prime$ selected at random from the eight, first- and second-order neighbors. We then calculate the change, Δ*H*, of the Hamiltonian *H* = *H*_contact_ + *H*_size_, which defines the force resulting from cell behaviors and properties in the model. An additional energy *H*_0_ is added to Δ*H* at the time of copying to represent dissipative energies (or other copy biases), including those associated with physical obstruction by the fibrin matrix. The components of the Hamiltonian and *H*_0_ are described in more detail below.

As in Hamiltonian systems $\vec{F}\propto \vec{\nabla }H$, any copy attempt for which Δ*H* + *H*_0_ < 0 represents a passive force (*e.g*., due to adhesion or pressure differences) that is sufficiently large to overcome the local, dissipative energies. These copy attempts are always accepted. In addition, cells exert active forces on their environment due to random membrane fluctuations; we assume these fluctuations are distributed according to the Boltzmann probability function,

$$P_{\text{Boltzmann}}\left(\Delta H,H_{0}\right)=e^{\frac{-\Delta H+H_{0}}{\mu }},$$ (1)

with *μ*, the rate of active random membrane fluctuations (a.k.a. cellular temperature).

The model includes a number of “static” cells, *τ*(*σ*) ∈ {cell patch, border}. Any copy attempt from or to the static states is ignored (*i.e*., updates are applied only if $\tau \left(\sigma \left(\vec{x}\right)\right)\in \left\{\text{medium},\text{cell}\right\}\wedge \tau \left(\sigma \left(\vec{x^{\prime }}\right)\right)\in \left\{\text{medium},\text{cell}\right\}$). Copy attempts from fibrin sites ($\tau (\sigma \left(\vec{x}\right))=\text{fibrin}$) are also ignored; copy attempts into fibrin ($\tau (\sigma \left(\vec{x}\prime \right))=\text{fibrin}$) are a special case (see Section Fibrin invasion).

#### Interfacial energies

The contact energy results from cell-cell and cell-fibrin adhesion and cortical tensions [77], and is defined as

$$H_{\text{interfacial}}=\sum_{\left(\vec{x},\vec{x}\prime \right)}J\left(\tau \left(\sigma \left(\vec{x}\right)\right),\tau \left(\sigma \left(\vec{x}\prime \right)\right)\right)(1-\delta (\sigma \left(\vec{x}\right),\sigma \left(\vec{x}\prime \right))).$$ (2)

The Kronecker delta (*δ*(*x*, *y*) = 1, for *x* = *y*, 0, for *x* ≠ *y*) restricts the interfacial energies to the cell membranes, and $\left(\vec{x},\vec{x}\prime \right)$ represents the set of all adjacent lattice site pairs in Λ. Each type combination has an interfacial energy *J*(*τ*, *τ*′), with low values representing stronger adhesion and lower cortical tension and high values weaker adhesion or repulsion and higher cortical tensions.

#### Cellular size constraints

To ensure that cells (*τ*(*σ*) = cell) stay close to their preferred size (*A*(*σ*)), we add a “volume” energy,

$$H_{\text{size}}=\sum_{s}\lambda_{A}(s){\left(A(s)-a(s)\right)}^{2},$$ (3)

with *a*(*s*) ≡ |*C*(*s*)|, the actual cell size (*i.e*., the number of lattice sites that the cell occupies), and λ_*A*_(*s*), a Lagrange multiplier representing inverse cell compressibility.

To keep cells connected, a large penalty (*H*_0_ = 1 × 10^9^) is added to the Hamiltonian for copy attempts that would break up a cell into disconnected patches.

#### Fibrin invasion

To model fibrin invasion, we coupled the CPM to a system of PDEs (see next Section) that describes all kinetic reactions involved in the uPAR-plasmin-TGF*β*1 feedback loop that we propose in this paper. The PDE concentration fields are discretized on a lattice of the same dimensions and spacing as the CPM lattice, such that, in addition to the cell identifier $\sigma (\vec{x})$, a set of chemical concentrations is associated with each lattice site $\vec{x}$.

The probability that a cell invades fibrin, thus performing an extending copy into fibrin, depends on the total concentration of fibrin at the invaded fibrin pixel ($f(\vec{x}\prime )$). The total concentration of fibrin is the sum of all the PDE components that contain fibrin,

$$f\left(\vec{x}\prime \right)=F\left(\vec{x}\prime \right)+F_{\text{PLG}}\left(\vec{x}\prime \right)+F_{\text{PLS}}\left(\vec{x}\prime \right)+F_{\text{LTGF}}\left(\vec{x}\prime \right)+F_{\text{PLG},\text{LTGF}}\left(\vec{x}\prime \right)+F_{\text{PLS},\text{LTGF}}\left(\vec{x}\prime \right).$$ (4)

To capture the reduced cell invasion into dense fibrin matrices, for fibrin densities $f\left(\vec{x}\prime \right)>\theta_{\text{fibrin}}$ we add to *H*_0_ (see Eq 1) an energy penalty of

$$\Delta H_{0,\text{invasion}}=\frac{p}{1+e^{-E(f\left(\vec{x}\prime \right)-m)}},$$ (5)

at the time of copying, with *p* = 1000, *E* = 10, *m* = 0.5, and *θ*_fibrin_ = 0.3. Thus sites with high fibrin concentration resist invasion by a pseudopod. These parameters where chosen in order to have a smooth transition from a small effect for low concentration to a saturation at high fibrin concentration, and to have an energy penalty of the same order of magnitude as the other terms on the Hamiltonian (the adhesion terms go from 15 to 120; the area constraint factor, the cell elasticity, is 100; the random membrane fluctuation value is 100). A sensitivity analysis for these parameters is given in S1 Fig and S1 Table.

#### Contact-inhibited mitosis

Every ten time steps, each cell has a probability (*P*_mitosis_) to divide over its short axis if it has little contact with other cells. More specifically, a cell may divide if *R*_*σ*_ < *R*_mitosis_, with $R_{\sigma }=\frac{\text{size}\;\text{membrane}\;\text{with}\;\text{cell}-\text{cell}\;\text{contact}}{\text{size}\;\text{total}\;\text{membrane}}$. The expression level of uPAR of the dividing cell is assigned to both daughter cells. With these division rules, the cell cycle has an average duration of 4.5 days for the default simulation parameters.

#### Quantification of the angiogenesis level

The angiogenesis level simultaneously reflects sprout depth and sprout count. At the end of each simulation, the angiogenesis level is calculated as follows: 1) Equally distributed horizontal lines, one per vertical lattice site, are drawn between 0 and 90% of initial fibrin matrix height. 2) For each line, the number of connected components consisting of cells or medium within fibrin are counted. Only the components larger than one cell size (20 lattice sites) and smaller than the complete line are counted. A component as large as the complete line would resemble lowering of the complete monolayer rather than sprouting. 3) The count of the lines is averaged.

### Plasminogen-plasmin system and uPAR-plasmin-TGF*β*1 positive feedback

**Fig 8:**
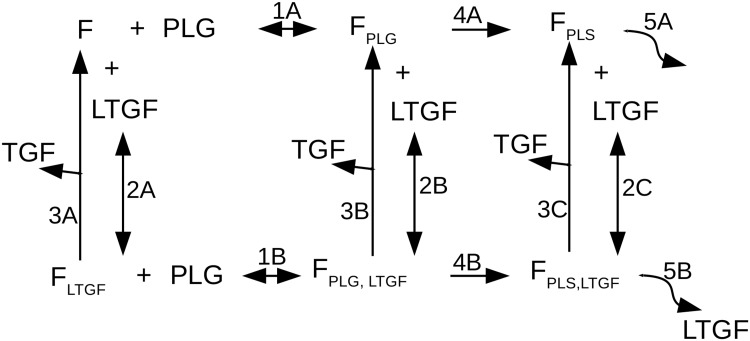
Overview of the binding and conversion reactions of plasminogen and latent-TGF*β*1 in relation to fibrin. Plasminogen (PLG) and latent-TGF*β*1 (LTGF) do not compete for binding with fibrin, thus fibrin can be unbound (F), bound solely by plasminogen (*F*_PLG_), bound by solely latent-TGF*β*1 (*F*_LTGF_), or by both (*F*_PLG,LTGF_). Plasminogen reversible binds fibrin (reactions 1A and 1B). Latent-TGF*β*1 also reversible binds fibrin (reactions 2A, 2B, and 2C). Latent-TGF*β*1 is released from fibrin by plasmin into the active form (TGF, reactions 3A, 3B, and 3C). Fibrin-bound plasminogen can be converted to fibrin-bound plasmin, either without (*F*_PLS_, reaction 4A) or with (*F*_PLS, LTGF_, reaction 4B) co-binding of latent-TGF*β*1. Reactions 5A and 5B represent fibrinolysis, which can result in the release of latent-TGF*β*1 (reaction 5B).

The plasminogen-plasmin system in this model is based on the continuum model by Diamond *et al*. [36]. We made some changes to make it suitable for our system and, most importantly, we included the uPAR-plasmin-TGF*β*1 positive feedback, simplified the implementation of fibrinolysis, and removed convective terms. Fig 8 shows an overview of the binding and conversion reactions of plasminogen and latent-TGF*β*1 in relation to fibrin that are included in our model. In this section we will discuss the reactions in Fig 8 to explain the PDE system that describes the plasminogen-plasmin system and the uPAR-plasmin-TGF*β*1 positive feedback loop.

#### Latent-TGF*β*1 and plasminogen bind fibrin

Plasminogen (PLG) reversibly binds fibrin (F), forming fibrin-bound plasminogen (*F*_PLG_). This reaction (reaction 1A in Fig 8) has a forward rate *k*_*f*1_ and a reverse rate *k*_*r*1_. Similarly, the reversible binding of latent-TGF*β*1 (LTGF) to fibrin (reaction 2A) depends on a forward rate *k*_*f*2_ and a reverse rate *k*_*r*2_. LTGF that is bound to fibrin (*F*_LTGF_) can be released and activated by plasmin-mediated proteolytic activity, resulting in active TGF*β*1 and free fibrin (reaction 3A). This reaction follows Michaelis-Menten kinetics with constants *k*_*u*2_ and *k*_*m*2_. The change in concentration of fibrin depends on PLG-Fibrin binding (1A), LTGF-Fibrin binding (2A) and release of TGF*β* (3A), following:

$$\begin{array}{ccc} \frac{\partial F}{\partial t} & = & \overset{\text{PLG}-\text{Fibrin}\;\text{binding}\;(1A)}{\overset{︷}{-\left[\text{PLG}\right]*F*k_{f1}+F_{\text{PLG}}*k_{r1}}}+\overset{\text{LTGF}-\text{Fibrin}\;\text{binding}\;(2A)}{\overset{︷}{F_{\text{LTGF}}*k_{r2}-\left[\text{LTGF}\right]*F*k_{f2}}}+\\ & & \overset{\text{TGF}\beta \;\text{release}\;\left(3A\right)}{\overset{︷}{\frac{F_{\text{LTGF}}*\left(F_{\text{PLS}}+F_{\text{PLS},\text{LTGF}}\right)*k_{u2}}{k_{m2}+F_{\text{LTGF}}}}}. \end{array}$$ (6)

For brevity we write *V* for all space and time dependent variables $V(\vec{x},t)$ in the partial-differential equations. Multisymbol variable names are surrounded by square brackets.

We assume that plasminogen and latent-TGF*β*1 do not compete with each other for the binding sites in fibrin. Consequently, the rates of the reversible binding reaction of PLG to fibrin are equal to the rates for PLG binding to fibrin to which LTGF is bound (*F*_LTGF_), thus reactions 1A and 1B in Fig 8 have the same rate constants. Plasminogen binding to *F*_LTGF_ forms *F*_PLG,TGFL_ (reaction 1B). PLG diffuses with diffusion coefficient *D*_PLG_ and decays with rate *ϵ*_PLG_. The change in concentration of plasminogen depends on PLG-Fibrin binding (1A), PLG-*F*_LTGF_ binding (1B), and its diffusion and decay, following:

$$\begin{array}{ccc} \frac{\partial [\text{PLG}]}{\partial t} & = & \overset{\text{Diffusion}}{\overset{︷}{D_{\text{PLG}}\nabla^{2}\left[\text{PLG}\right]}}-\overset{\text{Decay}}{\overset{︷}{\epsilon_{\text{PLG}}\left[\text{PLG}\right]}}+\overset{\text{PLG}-\text{Fibrin}\;(\text{un})\text{binding}\;(1A)}{\overset{︷}{F_{\text{PLG}}*k_{r1}-\left[\text{PLG}\right]*F*k_{f1}}}+\\ & & \overset{\text{PLG}-F_{\text{LTGF}}\;\left(\text{un}\right)\text{binding}\;\left(1B\right)}{\overset{︷}{F_{\text{PLG},\text{LTGF}}*k_{r1}-\left[\text{PLG}\right]*F_{\text{LTGF}}*k_{f1}}}. \end{array}$$ (7)

Similarly, the reversible binding of LTGF to fibrin has equal rates for fibrin to which plasminogen or plasmin is bound, *F*_PLG_ and *F*_PLS_ respectively. Thus, reactions 2A, 2B and 2C in Fig 8 have the same rate constants. Unbound LTGF diffuses with diffusion coefficient *D*_LTGF_ and decays with rate *ϵ*_LTGF_. LTGF is released from the matrix upon plasmin-mediated fibrinolysis (reaction 5B), modeled with a Hill equation with constant *d*. The change in concentration of latent-TGF*β*1 depends on LTGF-Fibrin binding (2A), LTGF-*F*_PLG_ binding (2B), LTGF-*F*_PLS_ binding (2C), release of LTGF (5B), and its diffusion and decay, following:

$$\begin{array}{ccc} \frac{\partial [\text{LTGF}]}{\partial t} & = & \overset{\text{Diffusion}}{\overset{︷}{D_{\text{LTGF}}\nabla^{2}\left[\text{LTGF}\right]}}-\overset{\text{Decay}}{\overset{︷}{\epsilon_{\text{LTGF}}\left[\text{LTGF}\right]}}+\overset{\text{LTGF}-\text{Fibrin}\;(\text{un})\text{binding}\;(2A)}{\overset{︷}{F_{\text{LTGF}}*k_{r2}-\left[\text{LTGF}\right]*F*k_{f2}}}+\\ & & \overset{\text{LTGF}-F_{\text{PLG}}\;\left(\text{un}\right)\text{binding}\;\left(2B\right)}{\overset{︷}{F_{\text{PLG},\text{LTGF}}*k_{r2}-\left[\text{LTGF}\right]*F_{\text{PLG}}*k_{f2}}}+\overset{\text{LTGF}\;\text{release}\;\left(5B\right)}{\overset{︷}{h\frac{F_{\text{PLS},\text{LTGF}}^{2}}{d+F_{\text{PLS},\text{LTGF}}^{2}}}}+\\ & & \overset{\left[\text{LTGF}\right]-F_{\text{PLS}}\;\left(\text{un}\right)\text{binding}\;\left(2C\right)}{\overset{︷}{F_{\text{PLS},\text{LTGF}}*k_{r2}-\left[\text{LTGF}\right]*F_{\text{PLS}}*k_{f2}}}. \end{array}$$ (8)

The change in concentration of fibrin-bound latent-TGF*β*1 depends on PLG-*F*_LTGF_ (un)binding (1B), LTGF-Fibrin (un)binding (2A), release of TGF (3A), and its decay, following:

$$\begin{array}{ccc} \frac{\partial F_{\text{LTGF}}}{\partial t} & = & -\overset{\text{Decay}}{\overset{︷}{\epsilon_{F_{\text{LTGF}}}F_{\text{LTGF}}}}+\overset{\left[\text{PLG}\right]-F_{\text{LTGF}}\;\left(\text{un}\right)\text{binding}\;\left(1B\right)}{\overset{︷}{F_{\text{PLG},\text{LTGF}}*k_{r1}-\left[\text{PLG}\right]*F_{\text{LTGF}}*k_{f1}}}+\\ & & \overset{\text{LTGF}-\text{Fibrin}\;(\text{un})\text{binding}\;(2A)}{\overset{︷}{\left[\text{LTGF}\right]*F*k_{f2}-F_{\text{LTGF}}*k_{r2}}}-\overset{\text{TGF}\beta \;\text{release}\;\left(3A\right)}{\overset{︷}{\frac{F_{\text{LTGF}}*\left(F_{\text{PLS}}+F_{\text{PLS},\text{LTGF}}\right)*k_{u2}}{k_{m2}+F_{\text{LTGF}}}}}. \end{array}$$ (9)

#### Plasminogen conversion into plasmin

Fibrin-bound plasminogen (*F*_PLG_) can be converted to fibrin-bound plasmin (*F*_PLS_). This conversion (reaction 4A) occurs in proximity of uPAR, modeled by Michaelis-Menten kinetics with rate constants *k*_*u*1_ and *k*_*m*1_. To express the proximity of uPAR, the expression level of uPAR at a certain pixel is calculated by taking the average expression level of uPAR of that pixel and of its first- and second-neighboring pixels (together forming the set of pixels NB_8_(*σ*)), resulting in *U* = < *uPAR*_*σ*_ >_NB_8_(*σ*)_. The change in concentration of fibrin-bound plasminogen depends on PLG-Fibrin binding (1A), LTGF-*F*_PLG_ binding (2B), plasmin activation (4A), release of TGF (3B) and its decay, following:

$$\begin{array}{ccc} \frac{\partial F_{\text{PLG}}}{\partial t} & = & \overset{\text{PLG}-\text{Fibrin}\;(\text{un})\text{binding}\;(1A)}{\overset{︷}{\left[\text{PLG}\right]*F*k_{f1}-F_{\text{PLG}}*k_{r1}}}-\overset{\text{Plasmin}\;\text{activation}\;(4A)}{\overset{︷}{\frac{F_{\text{PLG}}*U*k_{u1}}{k_{m1}+F_{\text{PLG}}}}}+\\ & & \overset{\text{LTGF}-F_{\text{PLG}}\;\left(\text{un}\right)\text{binding}\;\left(2B\right)}{\overset{︷}{F_{\text{PLG},\text{LTGF}}*k_{r2}-\left[\text{LTGF}\right]*F_{\text{PLG}}*k_{f2}}}-\overset{\text{Decay}}{\overset{︷}{\epsilon_{F_{\text{PLG}}}F_{\text{PLG}}}}+\\ & & \overset{\text{TGF}\beta \;\text{release}\;(3B)}{\overset{︷}{\frac{F_{\text{PLG},\text{LTGF}}*\left(F_{\text{PLS}}+F_{\text{PLS},\text{LTGF}}\right)*k_{u2}}{k_{m2}+F_{\text{PLG},\text{LTGF}}}}}. \end{array}$$ (10)

The change in concentration of *F*_PLG,LTGF_ depends on PLG-*F*_LTGF_ (un)binding (1B), LTGF-*F*_PLG_ (un)binding (2B), plasmin activation (4B), release of TGF (3B) and its decay, following:

$$\begin{array}{ccc} \frac{\partial F_{\text{PLG},\text{LTGF}}}{\partial t} & = & -\overset{\text{Decay}}{\overset{︷}{\epsilon_{F_{\text{PLG},\text{LTGF}}}F_{\text{PLG},\text{LTGF}}}}+\overset{\text{PLG}-F_{\text{LTGF}}\;\left(\text{un}\right)\text{binding}\;\left(1B\right)}{\overset{︷}{PLG*F_{\text{LTGF}}*k_{f1}-F_{\text{PLG},\text{LTGF}}*k_{r1}}}+\\ & & \overset{\left[\text{LTGF}\right]-F_{\text{PLG}}\;\left(\text{un}\right)\text{binding}\;\left(2B\right)}{\overset{︷}{\left[\text{LTGF}\right]*F_{\text{PLG}}*k_{f2}-F_{\text{PLG},\text{LTGF}}*k_{r2}}}-\overset{\text{Plasmin}\;\text{activation}\;\left(4B\right)}{\overset{︷}{\frac{F_{\text{PLG},\text{LTGF}}*U*k_{u1}}{k_{m1}+F_{\text{PLG},\text{LTGF}}}}}-\\ & & \overset{\text{TGF}\beta \;\text{release}\;\left(3B\right)}{\overset{︷}{\frac{F_{\text{PLG},\text{LTGF}}*\left(F_{\text{PLS}}+F_{\text{PLS},\text{LTGF}}\right)*k_{u2}}{k_{m2}+F_{\text{PLG},\text{LTGF}}}}} \end{array}$$ (11)

#### Plasmin activity

Fibrin-bound plasminogen is converted to fibrin-bound plasmin by uPAR-bound uPA. *F*_PLS_ is the conversion product of *F*_PLG_ (reaction 4A), and *F*_PLS,LTGF_ is the conversion product of *F*_PLG,LTGF_ (reaction 4B). To model fibrinolysis, *F*_PLS_ (reaction 5A) and *F*_PLS,LTGF_ (reaction 5B) are degraded, modeled with a Hill equation with constant *d*. The change in concentration of *F*_PLS_ depends on plasmin activation (4A), fibrinolysis (5A), LTGF-*F*_PLS_ binding (2C), release of TGF (3C), and its decay, following:

$$\begin{array}{ccc} \frac{\partial F_{\text{PLS}}}{\partial t} & = & -\overset{\text{Decay}}{\overset{︷}{\epsilon_{F_{\text{PLS}}}F_{\text{PLS}}}}+\overset{\text{Plasmin}\;\text{activation}\;\left(4A\right)}{\overset{︷}{\frac{F_{\text{PLG}}*U*k_{u1}}{k_{m1}+F_{\text{PLG}}}}}-\overset{\text{Fibrinolysis}\;\left(5A\right)}{\overset{︷}{h\frac{F_{\text{PLS}}^{2}}{d+F_{\text{PLS}}^{2}}}}+\\ & & \overset{\left[\text{LTGF}\right]-F_{\text{PLS}}\;\left(\text{un}\right)\text{binding}\;\left(2C\right)}{\overset{︷}{F_{\text{PLS},\text{LTGF}}*k_{r2}-\left[\text{LTGF}\right]*F_{\text{PLS}}*k_{f2}}}+\\ & & \overset{\text{TGF}\beta \;\text{release}\;\left(3C\right)}{\overset{︷}{\frac{F_{\text{PLS},\text{LTGF}}*\left(F_{\text{PLS}}+F_{\text{PLS},\text{LTGF}}\right)*k_{u2}}{k_{m2}+F_{\text{PLS},\text{LTGF}}}}}. \end{array}$$ (12)

The change in concentration of *F*_PLS,LTGF_ depends on plasmin activation (4B), fibrinolysis (5B), LTGF-*F*_PLS_ binding (2C), release of TGF (3C), and its decay, following:

$$\begin{array}{ccc} \frac{\partial F_{\text{PLS},\text{LTGF}}}{\partial t} & = & -\overset{\text{Decay}}{\overset{︷}{\epsilon_{F_{\text{PLS},\text{LTGF}}}F_{\text{PLS},\text{LTGF}}}}+\overset{\text{Plasmin}\;\text{activation}\;\left(4B\right)}{\overset{︷}{\frac{F_{\text{PLG},\text{LTGF}}*U*k_{u1}}{k_{m1}+F_{\text{PLG},\text{LTGF}}}}}-\\ & & \overset{\text{Fibrinolysis}\;\left(5B\right)}{\overset{︷}{h\frac{F_{\text{PLS},\text{LTGF}}^{2}}{d+F_{\text{PLS},\text{LTGF}}^{2}}}}+\overset{\left[\text{LTGF}\right]-F_{\text{PLS}}\;\left(\text{un}\right)\text{binding}\;\left(2C\right)}{\overset{︷}{\left[\text{LTGF}\right]*F_{\text{PLS}}*k_{f2}-F_{\text{PLS},\text{LTGF}}*k_{r2}}}-\\ & & \overset{\text{TGF}\beta \;\text{release}\;\left(3C\right)}{\overset{︷}{\frac{F_{\text{PLS},\text{LTGF}}*\left(F_{\text{PLS}}+F_{\text{PLS},\text{LTGF}}\right)*k_{u2}}{k_{m2}+F_{\text{PLS},\text{LTGF}}}}}. \end{array}$$ (13)

#### TGF*β*1 activation

Plasmin can release and activate latent-TGF*β*1, resulting in active TGF*β*1 (TGF) that diffuses with diffusion coefficient *D*_TGF_ and decays with rate *ϵ*_TGF_. TGF can originate from each form of fibrin-bound latent-TGF*β*1 (*F*_LTGF_, *F*_PLG,LTGF_ and *F*_PLS,LTGF_), released by plasmin following Michaelis-Menten kinetics with constants *k*_*u*2_ and *k*_*m*2_. The change in concentration of TGF depends on release of TGF (3A, 3B, and 3C), and its diffusion and decay, following:

$$\begin{array}{ccc} \frac{\partial [\text{TGF}]}{\partial t} & = & \overset{\text{Diffusion}}{\overset{︷}{D_{\text{TGF}}\nabla^{2}\left[\text{TGF}\right]}}-\overset{\text{Decay}}{\overset{︷}{\epsilon_{\text{TGF}}\left[\text{TGF}\right]}}+\overset{\text{TGF}\beta \;\text{release}\;\left(3A\right)}{\overset{︷}{\frac{F_{\text{LTGF}}*\left(F_{\text{PLS}}+F_{\text{PLS},\text{LTGF}}\right)*k_{u2}}{k_{m2}+F_{\text{LTGF}}}}}+\\ & & \overset{\text{TGF}\beta \;\text{release}\;\left(3B\right)}{\overset{︷}{\frac{F_{\text{PLG},\text{LTGF}}*\left(F_{\text{PLS}}+F_{\text{PLS},\text{LTGF}}\right)*k_{u2}}{k_{m2}+F_{\text{PLG},\text{LTGF}}}}}+\\ & & \overset{\text{TGF}\beta \;\text{release}\;\left(3C\right)}{\overset{︷}{\frac{F_{\text{PLS},\text{LTGF}}*\left(F_{\text{PLS}}+F_{\text{PLS},\text{LTGF}}\right)*k_{u2}}{k_{m2}+F_{\text{PLS},\text{LTGF}}}}} \end{array}$$ (14)

#### PAI-1 secretion

The cells secrete PAI-1 ([*PAI*]) at rate *α* at each site that they occupy. PAI diffuses with diffusion coefficient *D*_PAI_, it is degraded at a rate *ϵ*_PAI_, and it is taken up by the cells through binding with uPAR. PAI-1 binds uPAR-bound uPA for inactivation, resulting in a depletion of PAI and uPAR with rate *k*_*f*3_. For this purpose the concentration of PAI is calculated over all cell pixels (*C*_*σ*_). The change in concentration of PAI depends on its internalization when bound to uPAR, its secretion, and its diffusion and decay, giving:

$$\frac{\partial [\text{PAI}]}{\partial t}=\overset{\text{Diffusion}}{\overset{︷}{D_{\text{PAI}}\nabla^{2}\left[\text{PAI}\right]}}-\overset{\text{Decay}}{\overset{︷}{\epsilon_{\text{PAI}}\left[\text{PAI}\right]}}-\overset{\text{Internalization}}{\overset{︷}{\left[\text{uPAR}\right]\left(\vec{x},t\right)*\left[\text{PAI}\right]*k_{f3})}}+\overset{\text{Secretion}\;\text{at}\;\text{cells}}{\overset{︷}{\alpha 1_{\left\{\vec{x}\in \Lambda |\tau \left(\vec{x}\right)=\text{cell}\right\}}}}.$$ (15)

with *P*(*σ*), the perimeter of cell *σ*.

#### Activation and inhibition of uPAR

We assume that the uPA-receptor (uPAR) remains distributed uniformly over the cell membranes. Following this assumption, we represent the uPAR-concentrations by one ordinary-differential equation for each individual cell. We assume that the concentration of uPAR depends on a baseline production rate, *c*, and an additional production induced by TGF*β*. uPAR undergoes first order degradation; further degradation occurs due to internalization upon binding to PAI,

$$\begin{array}{ccc} \frac{d[\text{uPAR}](s)}{dt} & = & -\overset{\text{Decay}}{\overset{︷}{\epsilon_{\text{uPAR}}\left[\text{uPAR}\right]\left(s\right)}}-\overset{\text{Internalization}}{\overset{︷}{\sum_{\vec{x}\in C\left(s\right)}\left(\left[\text{uPAR}\right]\left(s\right)*\left[\text{PAI}\right]\left(\vec{x}\right)*k_{f3}\right)}}+\\ & & \overset{\text{Constant}\;\text{production}}{\overset{︷}{c}}+\overset{\text{TGF}-\text{induced}\;\text{uPAR}\;\text{production}}{\overset{︷}{\frac{k_{u3}{\left(\sum_{\vec{x}\in P\left(s\right)}\left[\text{TGF}\right]\left(\vec{x}\right)\right)}^{2}}{k_{m3}+{\left(\sum_{\vec{x}\in P\left(s\right)}\left[\text{TGF}\right]\left(\vec{x}\right)\right)}^{2}}}}, \end{array}$$ (16)

with [uPAR](*s*) the concentration of uPAR in cell *s*, and *P*(*s*) is the set of lattice sites at the boundary of cell *s* (*i.e*., $P\left(s\right)=\{\vec{x}\in C\left(s\right)|\left(\exists \vec{x}\prime \in {\text{NB}}_{8}\left(\vec{x}\right)\right)\left[\sigma \left(\vec{x}\right)\prime \neq s\right]\}$ with ${\text{NB}}_{8}\left(\vec{x}\right)$ the eight first and second nearest neighbors of $\vec{x}$). Prior to each Monte Carlo step the uPAR concentrations are copied to a field $\left[\text{uPAR}\right]\left(\vec{x},t\right)$ on a lattice coinciding with the CPM-grid.

#### Numerical implementation

The fibrin ingrowth model is implemented using CompuCell3D [78], using a combination of standard features and custom *Steppables* (Python modules to be performed once every Monte Carlo step) and *plug-ins* (C++ modules to be performed once every copy update). The simulation codes are in S1 Code.

The model couples a modified Cellular Potts model (CPM; Eqs 1–5) for cell motility, with ten partial-differential equations (PDE; Eqs 6–15) to describe fibrin and the plasmin system, and one ordinary-differential equation (ODE; Eq 16) for membrane-bound uPAR. The CPM, PDEs and ODE are coupled using an operator splitting approach—this approach alternates steps of the CPM, of the PDEs and the ODE. One time step proceeds as follows: we first perform one Monte Carlo step (MCS) of the CPM, while keeping the state of the PDEs and ODE fixed. During one MCS, as many copy attempts are performed as there are lattice sites in the grid, that is 500 × 150, with each lattice site representing 2 *μ*m × 2 *μ*m. Note that the CPM depends on the state of the PDEs (see Section Fibrin invasion, Eqs 4 and 5). We then freeze the state of the CPM and the PDEs, and perform one integration step of the ODE (Eq 16) using the explicit, forward Euler method with Δ*x* = 2 *μ*m and Δ*t* = 150 s. To facilitate interaction with the PDEs we make use of an auxiliary field $\left[\text{uPAR}\right]\left(\vec{x},t\right)$. After each ODE integration step the value [uPAR](*s*, *t*) is copied into all the sites of this uPAR field that the cell *s* occupies, such that $\forall \vec{x}\in C\left(s\right):\left[\text{uPAR}\right]\left(\vec{x},t\right):=\left[\text{uPAR}\right]\left(s,t\right)$. Concurrently with the ODE (*i.e*., based on $\left[uPAR\right]\left(\vec{x},t-\Delta t\right),$ but in practice after the ODEs in this sequential implementation), the PDEs are updated using an explicit forward Euler method. We apply 10 integration steps per MCS with Δ*t* = 15 s. This concludes one simulation time step.

Numerical stability was assured by keeping lattice diffusion coefficients below 0.25 and by checking for typical signs of numerical instability, including spurious oscillation in space and time, and run-offs to plus or minus infinity. No caps or other corrections were applied to constrain the numerical solutions.

#### Simulation set-up, boundary, and initial conditions

The model is initialized with a monolayer of fifty endothelial cells on top of a fibrin matrix and some medium on top. A high ‘border energy’ ensures that cells are repelled by the boundaries, except at the level of the cell monolayer. Here a small, immobile ‘cell patch’ is positioned in the border at the level of the monolayer to ‘glue’ the cell layer to the boundary thus mimicking a continuous monolayer of cells. The ‘cell patch’ is static, but otherwise has the same parameters as the regular cells.

The PDEs are initialized with a fibrin-bound concentration of latent-TGF*β*1 and plasminogen, by setting the initial concentration *F*_PLG,LTGF_ = 1 at every lattice site of type fibrin, *i.e*., $\forall \vec{x}\in \left\{\vec{x}\in \Lambda |\tau \left(\sigma \left(\vec{x}\right)\right)=\text{Fibrin}\right\}:F_{\text{PLG},\text{LTGF}}:=1.0$. All other concentrations are set relative to this concentration level, expressed in relative units (RU). For the model validation experiments, we either reduced the level of plasminogen or that of latent-TGF*β*1 in the fibrin matrix, and kept the total levels of plasminogen (PLG) and latent-TGF*β*1 constant. This was done by reducing the initial concentrations of *F*_PLG,LTGF_ or increasing the concentration of *F*_LTGF_ or *F*_PLG_, such that $\forall \vec{x}\in \left\{\vec{x}\in \Lambda |\tau \left(\sigma \left(\vec{x}\right)\right)=\text{fibrin}\right\}:F_{\text{PLG},\text{LTGF}}\left(\vec{x},0\right)+F_{\text{LTGF}}\left(\vec{x},0\right)=1$ and $F_{\text{PLG},\text{LTGF}}\left(\vec{x},0\right)+F_{\text{PLG}}\left(\vec{x},0\right)=1$

Sprouts fully develop in 6000 MCS in our model. Endothelial cells are cultured for 10 days [1], thus one MCS is equivalent to approximately 2.5 minutes. The parameter settings for the CPM are listed in Table 1. Except for cell size (*A*), the parameters of the cellular Potts model can only be qualitatively coupled to experimental data. The parameter settings for the ODEs and PDEs are listed in S2 Table.

**Table 1:** CPM parameters.

*A* = 800 *μ*m^2^	λ_*A*_ = 100	*μ* = 100
*J*_cell,Medium_ = 30	*J*_cell,cell_ = 15	*J*_fibrin,Medium_ = 120
*J*_cell,border_ = 1 × 10^6^	*J*_cell,fibrin_ = 75	*H*_connectivity_ = 1 × 10^9^
*P*_mitosis_ = 0.6	*R*_mitosis_ = 0.35	Δ*x* = 2 *μ*m
lattice dimensions = 1000 *μ*m ×300 *μ*m		

#### In vitro tube formation assay

3D human tube formation was evaluated as previously described [1]. 2 mg/ml fibrinogen (Stago bnl, Leiden, The Netherlands) was dissolved in M199 medium + p/s. Thrombin (0.05 U/ml) was added to the fibrinogen solution and 100μ*l* was immediately added to wells of a 96-well plate. For polymerization, plates were incubated for one hour at room temperature followed by one hour at 37°C. Thrombin was inactivated by addition of serum-supplemented culture (SSC) medium consisting of Medium 199 with p/s supplemented with 10% HSi, 10% NBCSi and 2 mM L-glutamine. hMVECs were seeded in a confluent density on top of the fibrin matrices in SSC medium. After 24 hours, and subsequently at 48h intervals, the hMVECs were stimulated with SSC medium supplemented with 10 ng/ml tumor necrosis factor-*α* (TNF*α*, ReliaTech GmbH, Wolfenbuttel, Germany) and 10 ng/ml fibroblast growth factor-2 (FGF-2, ReliaTech GmbH) in the absence or presence of 100 U/ml trasylol (Pentapharm Ltd., Basel, Switzerland), 25 *μ*g/ml anti-uPAR antibody H2 (gift of Dr. U Weidle, Boehringer Mannheim), control mIgG or anti-PAI-1 antibody MAI-2 (Biopool, Umeå, Sweden). The experiments were terminated after 7 days by fixation with 2% paraformaldehyde/HBSS for two hours at room temperature. The formation of tube-like structures from hMVECs into the three-dimensional fibrin matrices were analyzed using a phase contrast microscope and two-dimensional top views of the cell culture, making the endothelial tubes well visible [1]. The total length of tube-like structures of four randomly chosen microscopic fields/well (7.3 mm^2^/field) were measured using a Nikon FXA microscope equipped with a monochrome CCD camera (MX5) connected to a computer with Optimas image analysis software, and expressed as mean tube length of 4 independent donors ± standard error of the mean.

## Supporting information

S1 Video Sprouting by the uPAR-plasmin-TGF*β*1 positive feedback. This movie shows the selection of ingrowth spots in the endothelial cells monolayer and further sprouting due to the uPAR-plasmin-TGF*β*1 positive feedback in a simulation with default parameter settings. The movie is divided into four panels. The top left panel shows the CPM representation of cells (blue) and fibrin (yellow). The top right panel shows the expression of uPAR on the cells. The bottom left frame shows the concentration of active TGF*β*1 and the bottom right panel shows the concentration of PAI-1. Protein concentrations are colored according to a color bar, with red indicating the highest concentration in the current field and blue the lowest concentration.
(MP4)

S1 FigRepresentative simulations of a parameter sensitivity analysis of the effect of Eq 5 (obstruction of invasion by fibrin).State after 6000 MCS. (A) *p* = 500; (B) *p* = 2000; (C) *E* = 5; (D) *E* = 20; (E) *m* = 0.4; (F) *m* = 0.6; (G) *θ*_fibrin_ = 0.2; (H) *θ*_fibrin_ = 0.4; (I) default parameter set (*p* = 1000, *E* = 10, $m=\frac{1}{2}$, and *θ*_fibrin_ = 0.3).(PDF)

S2 Fig Independent replicates of Fig 1C–1E. Representative top views of vascular ingrowth in (A) unfragmented fibrin; (B) in HMW fibrin; and (C) in LMW fibrin. Experiments were performed as previously described [1]. Bars represent 500 *μ*m.
(PDF)

S1 Table Parameter sensitivity analysis of the effect of Eq 5. *n* = 10, ranges show standard deviation.
(PDF)

S2 Table Default (qualitative) parameter settings for fibrin model. The concentrations of all proteins are expressed in relative units (RU); one MCS represents approximately 2.5 minutes.
(PDF)

S1 Code ZIP file containing the model implementation in *CompuCell3D* [78]. The ZIP file includes custom Steppables (Python) and Plugins (C++), installation instructions, and a brief documentation. The open source package CompuCell3D (‘DeveloperZone’) must be installed separately from http://CompuCell3D.org.
(ZIP)
