# Supplementary figures and images for: A local uPAR-plasmin-TGFβ1 positive feedback loop in a qualitative computational model of angiogenic sprouting explains the in vitro effect of fibrinogen variants
Source: PLoS Comput Biol. 2018 Jul 6;14(7):e1006239. doi: 10.1371/journal.pcbi.1006239 (PMC6072121; doi:10.1371/journal.pcbi.1006239)

A

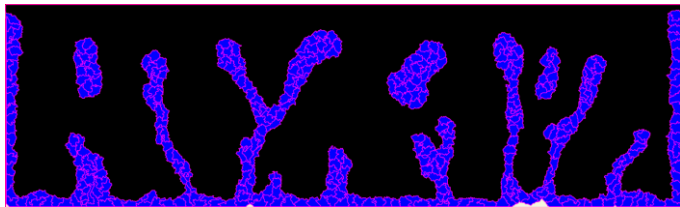

B

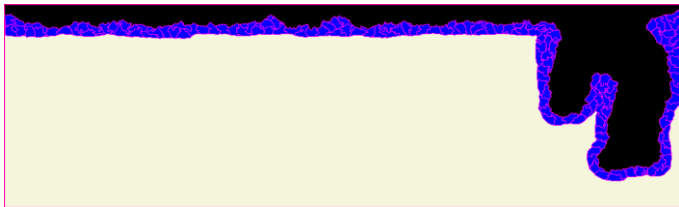

C

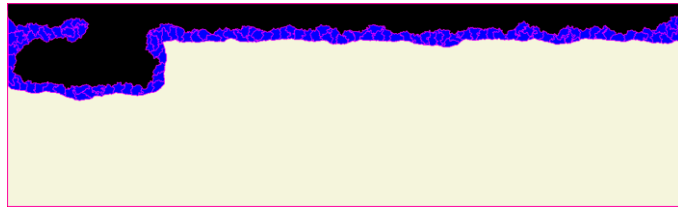

D

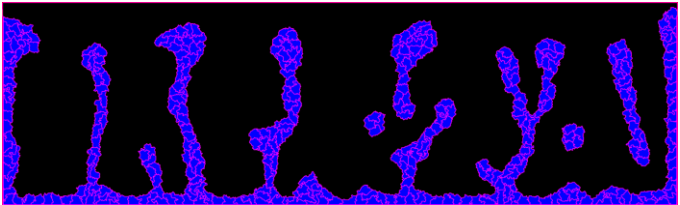

E

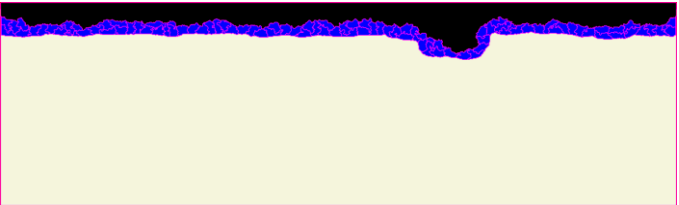

F

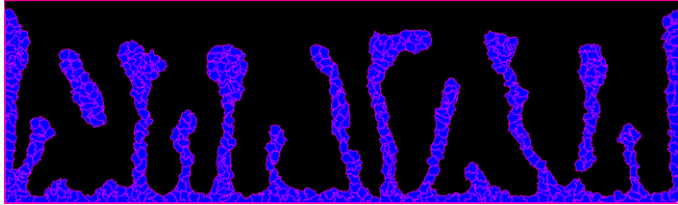

G

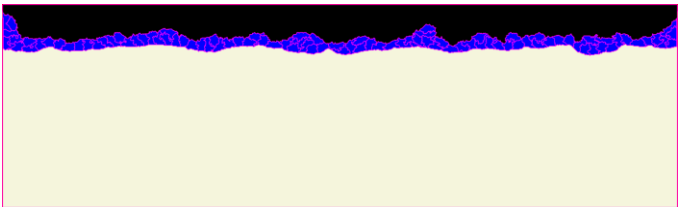

H

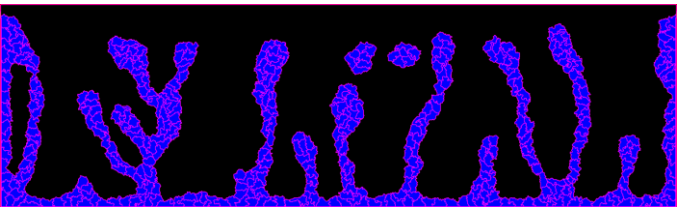

I

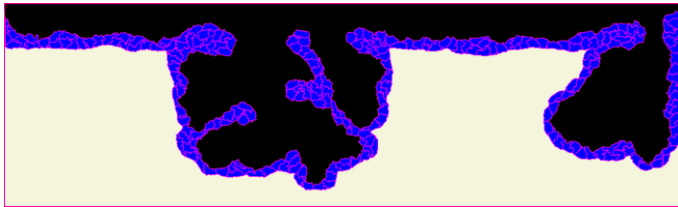

Supplement: S1 Fig — State after 6000 MCS. (A) p = 500; (B) p = 2000; (C) E = 5; (D) E = 20; (E) m = 0.4; (F) m = 0.6; (G) θfibrin = 0.2; (H) θfibrin = 0.4; (I) default parameter set (p = 1000, E = 10, m=12, and θfibrin = 0.3). (PDF) [file pcbi.1006239.s002.pdf]

**A**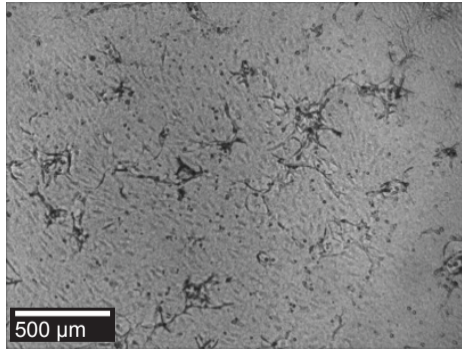**B**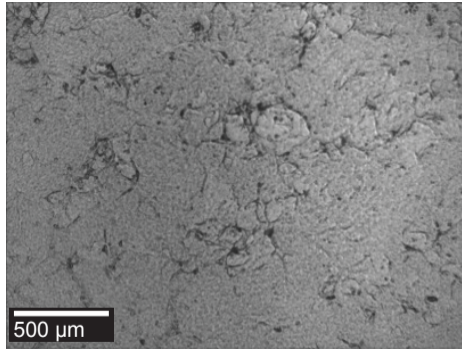**C**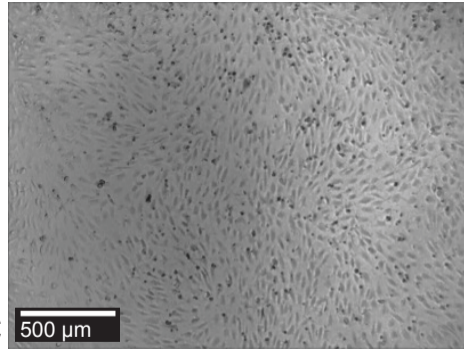

Supplement: S2 Fig — Representative top views of vascular ingrowth in (A) unfragmented fibrin; (B) in HMW fibrin; and (C) in LMW fibrin. Experiments were performed as previously described [1]. Bars represent 500 μm. (PDF) [file pcbi.1006239.s003.pdf]
