# Supplementary material for: A local uPAR-plasmin-TGFβ1 positive feedback loop in a qualitative computational model of angiogenic sprouting explains the in vitro effect of fibrinogen variants
Source: PLoS Comput Biol. 2018 Jul 6;14(7):e1006239. doi: 10.1371/journal.pcbi.1006239 (PMC6072121; doi:10.1371/journal.pcbi.1006239)
Supplement: S1 Table — n = 10, ranges show standard deviation. (PDF) [file pcbi.1006239.s004.pdf]

Difference from default	Angiogenesis level	% fibrinolysis	sprouting percentage
-	1.68 ± 0.2	56.2 ± 10.3	384 ± 108
$\theta_{\mathrm{fibrin}} = 0.4$	-	-	
$\theta_{\mathrm{fibrin}} = 0.2$	1.2 ± 0.2	31.0 ± 9.3	249 ± 104
$E = 5$	1.3 ± 0.3	26.9 ± 13.1	248 ± 158
$E = 20$	1.0 ± 0.0	99.1 ± 0.8	7.5 ± 3.5
$m = 0.4$	0.9 ± 0.4	17.6 ± 8	170 ± 124
$m = 0.6$	1.08 ± 0.12	99.40 ± 0.05	5.3 ± 4.8
$p = 2000$	0.58 ± 0.31	11.44 ± 4.61	79 ± 89
$p = 500$	1.19 ± 0.19	99.33 ± 0.19	14.5 ± 1.5
