# Supplementary material for: A local uPAR-plasmin-TGFβ1 positive feedback loop in a qualitative computational model of angiogenic sprouting explains the in vitro effect of fibrinogen variants
Source: PLoS Comput Biol. 2018 Jul 6;14(7):e1006239. doi: 10.1371/journal.pcbi.1006239 (PMC6072121; doi:10.1371/journal.pcbi.1006239)
Supplement: S2 Table — The concentrations of all proteins are expressed in relative units (RU); one MCS represents approximately 2.5 minutes. (PDF) [file pcbi.1006239.s005.pdf]

Parameter	Symbol	Value with Unit	Motivation
Initial value for fibrin with bound plasminogen and co-bound latent-TGFβ1	$F_{\mathrm{PLG,LTGF}}(t=0)$	1	fraction
Initial values for uPA receptor	$\mathrm{uPAR}(t=0)$	1 RU	
Diffusion rate of plasminogen	D_{PLG}	5×10^{-12} m^2/MCS	
Diffusion rate of latent TGFβ1	D_{LTGF}	5×10^{-12} m^2/MCS	
Diffusion rate of TGFβ1	D_{TGF}	5×10^{-12} m^2/MCS	
Diffusion rate of PAI-1	$D_{[\mathrm{PAI}]}$	5×10^{-12} m^2/MCS	
Michaelis-Menten constant	k_{m1}	1 RU	
Michaelis-Menten constant	k_{m2}	1.1 RU	
Michaelis-Menten constant	k_{m3}	8 RU^2	
Michaelis-Menten constant for fibrinolysis	d	4×10^{-2} RU^2	
Degradation rate of fibrin-plasminogen complex	$\epsilon_{\mathrm{F_{PLG}}}$	1×10^{-4} MCS^{-1}	estimated
Degradation rate for fibrin-bound plasmin	$\epsilon_{F_{\mathrm{PLS}}}$	1×10^{-4} MCS^{-1}	estimated
Degration rate of fibrin-bound latent TGFβ1	$\epsilon_{F_{\mathrm{LTGF}}}$	1×10^{-4} MCS^{-1}	estimated
Degradation rate of fibrin with bound plasminogen and co-bound latent-TGFβ1	$\epsilon_{F_{\mathrm{PLG,LTGF}}}$	1×10^{-4} MCS^{-1}	
Degradation rate of fibrin with bound plasmin and co-bound latent-TGFβ1	$\epsilon_{F_{\mathrm{PLS,LTGF}}}$	1×10^{-4} MCS^{-1}	
Degradation rate of PAI-1	ϵ_{PAI}	1×10^{-2} MCS^{-1}	
Degradation rate of TGFβ1	ϵ_{TGF}	5×10^{-2} MCS^{-1}	
Degradation rate for uPA receptor	ϵ_{uPAR}	9.5×10^{-3} MCS^{-1}	
Degradation rate of plasminogen	ϵ_{PLG}	1×10^{-3} MCS^{-1}	
Degradation rate of Latent TGFβ1	ϵ_{LTGF}	1×10^{-3} MCS^{-1}	
Secretion rate of PAI-1	α	0.01 RU/MCS	estimated
Rate constant for fibrinolysis	h	1 RU/MCS	
Production rate of uPA receptor	c	5×10^{-3} RU/MCS	
Binding rate of plasminogen to fibrin-latent-TGFβ1 complex	k_{f1}	1×10^{-2} MCS^{-1}RU^{-1}	
Unbinding rate of plasminogen from latent-TGFβ1 complex	k_{r1}	1×10^{-4} MCS^{-1}	
Binding rate of latent TGFβ1 to fibrin-plasmin complex	k_{f2}	1×10^{-2} MCS^{-1}RU^{-1}	
Unbinding rate of latent TGFβ1 from fibrin-plasmin complex	k_{r2}	1×10^{-8} MCS-1	
Rate of PAI internalization after binding to uPA receptor	k_{f3}	1×10^{-2} MCS^{-1}RU^{-1}	
Activation rate of fibrin-bound plasminogen	k_{u1}	1 MCS^{-1}	
Rate of TGFβ1 release from fibrin due to plasmin	k_{u2}	0.8 MCS^{-1}	
TGFβ1-induced uPA receptor production rate	k_{u3}	0.85 RU/MCS	

S2 Table Default parameter settings. The concentration of all proteins is expressed in relative units (RU) and one MCS represents approximately 2.5 minutes.
