# Supplementary material for: A local uPAR-plasmin-TGFβ1 positive feedback loop in a qualitative computational model of angiogenic sprouting explains the in vitro effect of fibrinogen variants
Source: PLoS Comput Biol. 2018 Jul 6;14(7):e1006239. doi: 10.1371/journal.pcbi.1006239 (PMC6072121; doi:10.1371/journal.pcbi.1006239)
Supplement: S1 Code — The ZIP file includes custom Steppables (Python) and Plugins (C++), installation instructions, and a brief documentation. The open source package CompuCell3D (‘DeveloperZone’) must be installed separately from http://CompuCell3D.org. (ZIP) [file pcbi.1006239.s006.zip › software/Doc_fibrin1.pdf]

A Local uPAR-plasmin-TGFβ1 Positive Feedback Loop in a Computational Model of Angiogenic Sprouting Explains the In Vitro Effect of Fibrinogen Variants

Sonja E.M. Boas, Joao Carvalho, Marloes van den Broek, Ester M. Weijers, Marie-José Goumans, Pieter Koolwijk, Roeland M.H. Merks

Documentation on the Fibrin model

Principle of the model:

To study by what mechanism the level of angiogenesis is reduced on LMW compare to HMW fibrin, we developed a computational model that mimics the in vitro assay by Koolwijk . The hybrid, cell-based and continuum computational model represents a cross-section of the in vitro model. The model is initialized with a mono layer of fifty endothelial cells on top of a fibrin matrix. Fibrin forms a physical obstruction for cells, while at the same time, fibrin offers cells support as cells can adhere to fibrin. Using cell-based modeling, we explicitly model cell shape, cell motility, cell-cell adhesion, and cell-fibrin adhesion. Each cell has a concentration of active uPAR homogeneously spread over its membrane and each cell secretes PAI-1. PAI-1, fibrin, latent-TGFβ1, active TGFβ1, plasminogen and plasmin are modeled as concentration fields and interact with each other, resulting in a local activation of plasminogen by cell-bound uPAR into plasmin. This releases and activates latent-TGFβ1 and degrades fibrin. Active TGFβ1 induces the production of uPAR in nearby cells, resulting in a local positive feedback loop that drives fibrin degradation. Cells can invade regions where fibrin is degraded, driven by adhesion of cells to the fibrin and contact-inhibited cell division.

To represent cells and their physical interactions with the fibrin matrix, the cellular Potts model was used. Cells are projected as patches of lattice sites on a lattice and move by copying lattice sites inward or outward, representing the extension and retraction of filopodia. A time step in the model, also called Monte Carlo step (MCS), represent approximately 2.5 minutes and a simulation takes 10 days similar to Koolwijk's assay. The concentration of uPAR for each cell is modeled by one ordinary differential equation (ODE). A concentration field for uPAR is projected on the CPM grid, with each lattice site that is occupied by a cell having the uPAR concentration of that cell. The concentration of uPAR moves along with the location of the cell after cell movement. A system of coupled partial differential equations describes the reactions between fibrin, TGFβ1, plasminogen, plasmin, PAI-1 and all fibrin-bound forms. The plasminogen-plasmin system in this model is based on the cell-free model by Diamond that studies the penetration of uPA and tPA in a fibrin clod present in the blood stream. To make it suitable for our question, we include the uPAR-plasmin-TGFβ1 positive feedback, simplified the implementation of fibrinolysis, and removed blood flow.

Software:

The model is developed in the CC3D modeling environment.
To run the model, first you need to compile the developer zone for this project:
../Simulations/Developerzone

Then you can run the XML: ../Simulations/XMLs/fibrinolysis_PLG_fibrin_noTip_large.xml
For the initial CPM cell configuration, it will load: ../Simulations/init_cells.pif

Analysis:

To make morphospaces, use: ../Analysis/morphospaceMovie_par_vs_par_seed_fibrin.py

To quantify sprouting, we used three measures: the angiogenesis level, the sprouting frequency and the fibrinolysis level: ../Analysis/run_xml_im_lisa_fibrin_analysis_home.py

- The angiogenesis level simultaneously reflects sprout depth and sprout count. At the end of each simulation, the angiogenesis level is calculated as follows: 1) Equally distributed horizontal lines are drawn, one per line, between 0 and 90 percent of initial fibrin matrix height. 2) For each line, the number of connected components consisting of cells or medium within fibrin are counted. Only the components larger than one cell size (20 lattice sites) and smaller than the complete line, which would resemble lowering of the complete mono layer rather than sprouting, are counted. 3) The count of the all lines is averaged.
- The sprouting frequency is the number of simulations out of one hundred simulations that formed sprouts.
- The fibrinolysis level, quantified as the mean percentage of initial fibrin lattice sites that are invaded by the endothelial cells in all one hundred simulations.

XML

The XML used for this project is: ../Simulations/XMLs/fibrinolysis_PLG_fibrin_noTip_large.xml

<Plugin Name="CellType">

In this simulation, we only use types medium, cell, fibrin, border and BM-3 functions as the cell-patch in the border at the height of the mono layer. The other types are still there because some are required to make the steppables <Plugin Name="ClusterDataTrackerPlugin"> and <Steppable Frequency="1" Type="MembranePolarizationFluidSteppable"> work. We use these plugins because we put a lot of extra functionalities in these plugins for cells. For instance, each cell has a concentration of uPAR, this is stored in the ClusterDataTrackerPlugin.

<Plugin Name="ClusterDataTrackerPlugin">

Cells are stored in a list with clusterIds and each Id has many properties, such as a volume, a list of cell compartments and protein concentration (e.g. uPAR).
This is what we use to store the uPAR concentration per cell: double uPARConcentration;
In this cluster is also the penalty for fibrin invasion for concentrations of fibrin:
code in: if (fibrinFlipPenalty){}

<Plugin Name="ClusterVolumePlugin">

This is the volume constraint for the clusterIds. The volume target value is initialized in <Plugin Name="ClusterDataTrackerPlugin"> by the volume at initialization.

<Steppable Frequency="1" Type="MembranePolarizationFluidSteppable">

We only use the init function of this steppable to activate the <Plugin Name="ClusterDataTrackerPlugin"> in which all clusters are initialized.
We skip the rest of the steppable by the use of:
<initializingStep>10000</initializingStep>

<Steppable Type="CellDivisionSteppable" Frequency="10">

Here we do contact-dependent division. We first measure which fraction of the cell membrane is in contact with other cells. If this fraction is below the minThreshold, then a cell has a

probability to divide (`divisionProbability`). The uPAR concentration of that cell is equally divided over the daughter cells.

`<Steppable Type="ReactionDiffusionSolverFE" Frequency="10000">`
To visualize the uPAR concentrations in the cells in the CC3D Gui, we use a field: `uPARact`
The steppable `<Steppable Type="TCSDrawFieldsSteppable" Frequency="10">` will update this field by assigning the uPAR concentration of a cell to each pixel that belongs to that cell.

`<Steppable Type="PLGsolverSteppable" Frequency="1">`
This is the most important steppable and performs all reactions between the fields.

In the start function of this steppable, we will first initialize the concentrations of all the fields (Fibrin, FPLG, FPLGLTGF, TGF etc)
`<TGFpresent>1</TGFpresent>` makes sure that all things with TGF are activated. Also it makes sure that all cells are initialized with the `InituPAR`:
`clusterDataTrackerPlugin->setuPARConcentration(ownClusterId,inituPAR)`. If `TGFpresent=0`, you will initialize with one tip cell that has uPAR

Then in the step:
`if (currentStep%1200==0){}` is for resetting the concentration of TGF to the initial value every 1200 MCS, we did this for extra experiment that mimics Pepper 1993.

In `if (currentStep%1==0)`, we get the boundary pixels of each cell and calculate the TGF concentration to upregulate uPAR production.

Then we go to `void PLGsolverSteppable::diffuse()`
this calls `solveRDEquations();` and `void PLGsolverSteppable::secrete()` as many times as indicated `by <ExtraTimesPerMCS>9</ExtraTimesPerMCS>` to solve the equations in smaller steps.

`PLGsolverSteppable::secrete()` simply does the secretion of PAI

`solveRDEquations();` calls `solvePLGTGFequations(idx`;, and this is where all the fields are solved.
There are four proteins that diffuse (PLG, PAI, TGF, TGFL), the rest is static in position.
We iterate over all pixels in the grid.
We first get all the current Field concentrations.
Then we get the concentration of uPAR in the pixel and its second neighbors, this will be used as uPAR concentration in the reactions.
Then we do diffusion for the four fields
Then we do all kinetic reactions
Then we store the new concentrations for all the fields.

`<Steppable Frequency="10" Type="SaveCellField">`
This steppable saves the sigma and tau and also for the fields that you specify.
